# Integrating the underlying structure of stochasticity into community ecology

**DOI:** 10.1002/ecy.2922

**Published:** 2019-12-26

**Authors:** Lauren G. Shoemaker, Lauren L. Sullivan, Ian Donohue, Juliano S. Cabral, Ryan J. Williams, Margaret M. Mayfield, Jonathan M. Chase, Chengjin Chu, W. Stanley Harpole, Andreas Huth, Janneke HilleRisLambers, Aubrie R. M. James, Nathan J. B. Kraft, Felix May, Ranjan Muthukrishnan, Sean Satterlee, Franziska Taubert, Xugao Wang, Thorsten Wiegand, Qiang Yang, Karen C. Abbott

**Affiliations:** ^1^ Department of Botany University of Wyoming 1000 E. University Ave. Laramie Wyoming 82017 USA; ^2^ Department of Ecology, Evolution, and Behavior University of Minnesota 1987 Upper Buford Circle Saint Paul Minnesota 55108 USA; ^3^ Department of Ecology and Evolutionary Biology University of Colorado 1900 Pleasant Street Boulder Colorado 80309 USA; ^4^ Division of Biological Sciences University of Missouri 105 Tucker Hall Columbia Missouri 65211 USA; ^5^ Department of Zoology, School of Natural Sciences Trinity College College Green Dublin 2 Ireland; ^6^ Synthesis Centre of the German Centre for Integrative Biodiversity Research (sDiv) Halle-Jena-Leipzig Deutscher Platz 5e Leipzig 04103 Germany; ^7^ Ecosystem Modeling, Center of Computation and Theoretical Biology University of Würzburg Emil-Fischer-Strasse 32 97074 Würzburg Germany; ^8^ The University of Queensland School of Biological Sciences Goddard Building Brisbane Queensland 4072 Australia; ^9^ German Centre for Integrative Biodiversity Research (iDiv) Deutscher Platz 5e Leipzig 04103 Germany; ^10^ Institute for Computer Science Martin Luther University Halle-Wittenberg Halle 06099 Germany; ^11^ Department of Ecology, State Key Laboratory of Biocontrol and School of Life Sciences Sun Yat-sen University 510275 Guangzhou Guangdong China; ^12^ Helmholtz Center for Environmental Research–UFZ Permoserstrasse 15 04318 Leipzig Germany; ^13^ Institute of Biology Martin Luther University Halle-Wittenberg Am Kirchtor 1 06108 Halle (Saale) Germany; ^14^ Institute of Environmental Research Systems University of Osnabrück P.O. Box 44 69,49069 Osnabrück Germany; ^15^ Department of Biology University of Washington Box 351800 Seattle WA 98195 USA; ^16^ Department of Ecology and Evolutionary Biology Cornell University E145 Corson Hall Ithaca New York 14853 USA; ^17^ Department of Ecology and Evolutionary Biology University of California, Los Angeles 621 Charles E. Young Drive East, P.O. Box 957246 Los Angeles CA 90095 USA; ^18^ Center for Methodology Leuphana University Lüneburg Universitätsallee 1 D‐21335 Lüneburg Germany; ^19^ Environmental Resilience Institute Indiana University 717 E 8th St Bloomington Indiana 47408 USA; ^20^ Department of Fisheries, Wildlife, and Conservation Biology University of Minnesota 2003 Upper Buford Circle St. Paul Minnesota 55108 USA; ^21^ Department of Ecology, Evolution, and Organismal Biology Iowa State University 251 Bessey Hall Ames Iowa 50011 USA; ^22^ CAS Key Laboratory of Forest Ecology and Management, Institute of Applied Ecology Chinese Academy of Sciences Shenyang 110016 China; ^23^ Department of Biology University of Konstanz Universitätsstraße 10 78464 Konstanz Germany; ^24^ Department of Biology Case Western Reserve University 10900 Euclid Avenue Cleveland OH 44106 USA

**Keywords:** autocorrelation, demographic stochasticity, distribution, diversity, environmental stochasticity, population dynamics, scale, uncertainty

## Abstract

Stochasticity is a core component of ecology, as it underlies key processes that structure and create variability in nature. Despite its fundamental importance in ecological systems, the concept is often treated as synonymous with unpredictability in community ecology, and studies tend to focus on single forms of stochasticity rather than taking a more holistic view. This has led to multiple narratives for how stochasticity mediates community dynamics. Here, we present a framework that describes how different forms of stochasticity (notably demographic and environmental stochasticity) combine to provide underlying and predictable structure in diverse communities. This framework builds on the deep ecological understanding of stochastic processes acting at individual and population levels and in modules of a few interacting species. We support our framework with a mathematical model that we use to synthesize key literature, demonstrating that stochasticity is more than simple uncertainty. Rather, stochasticity has profound and predictable effects on community dynamics that are critical for understanding how diversity is maintained. We propose next steps that ecologists might use to explore the role of stochasticity for structuring communities in theoretical and empirical systems, and thereby enhance our understanding of community dynamics.

## Introduction

Variability plays a central role in structuring ecological communities (Chesson 2000, Ives and Carpenter 2007, Leibold and Chase 2018). This variability comes from multiple sources, such as heterogeneity across space and time (Tilman 1994, Mouquet and Loreau 2003, Questad and Foster 2008, Hart et al. 2017), variation among individuals (Clark 2010, Bolnick et al. 2011, Clark et al. 2011), and stochasticity (Lande 1993, Vellend et al. 2014, Vellend 2016). Although stochasticity is well known to be one of the key sources of variability in ecological communities, it is not considered as a driver of community dynamics as frequently as other forms of variability (Hart et al. 2017). For example, in diverse communities stochasticity is often equated with neutrality (Vellend et al. 2014) or is simply treated as an impediment to our ability to understand dynamics (Boettiger 2018). In fact, stochasticity arises from the probabilistic nature of core biological processes, including births, deaths, species interactions, and movement (May 1973, Cohen 1976, Clark 2005, Black and McKane 2012), each of which can be described by an underlying distribution of possible events. Communities are the outcome of interactions among these biological processes. This implies that stochasticity plays an overarching and critical role in determining the structure and function of communities (May 1973, Vellend 2016).

Biological processes by their very nature are probabilistic, and thus stochastic. For a given biological process, repeated sampling from the same underlying probability distribution results in inherent variation between observed individual random outcomes (see Box 1 for a comprehensive description; Appendix S1 for definitions). The use of the term *stochasticity* often differs across studies and subfields (Hart et al. 2017; Appendix S2). These differences in terminology impede a synthetic understanding of how stochasticity shapes ecological dynamics, often leaving readers with the impression that the effects of stochasticity are idiosyncratic. These inconsistencies perpetuate misconceptions that stochasticity can be dismissed as unexplained variance, noise, or fundamental unpredictability. To address this, we first present a conceptual and modeling framework that unifies classic examples from the population dynamics literature (May 1973, Lande 1993, Boettiger 2018). We then extend our unifying framework to explore the impact of stochasticity on the dynamics of diverse communities (e.g., extending from 2–3 to 20 species). Using our framework, we move beyond simply acknowledging that stochasticity is important in community ecology, and focus on developing a more synthetic understanding of how multiple forms of stochasticity, in isolation and in combination, lead to predictable community‐level outcomes.

**Figure 1:**
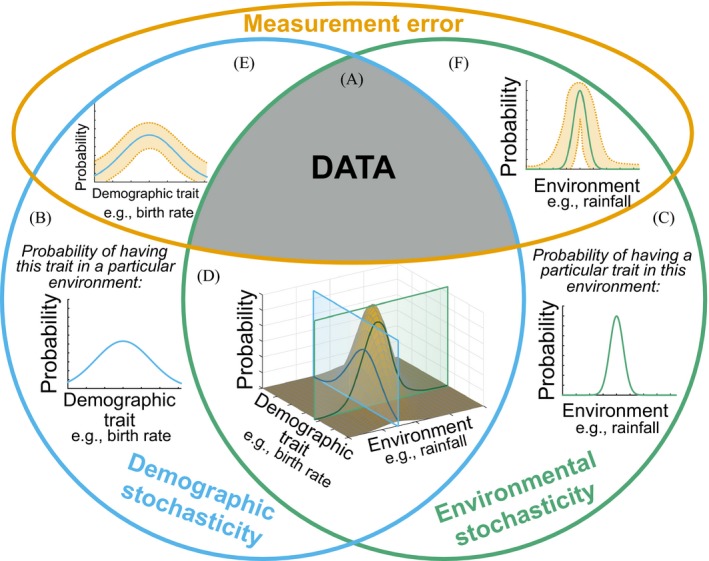
The three forms of stochasticity and how they influence observed population and community data. Data observations (A) are influenced by stochasticity in three general forms: demographic (e.g., stochasticity in intrinsic processes such as birth and deaths), environmental (e.g., stochasticity in extrinsic environmental conditions such as rainfall and temperature), and measurement error (e.g., imprecise data collection). Both demographic (B) and environmental (C) stochasticity represent biologically meaningful forms of uncertainty. Organisms in the environment experience these forms of stochasticity simultaneously (D), resulting in a multidimensional distribution of vital rates. Measurement error increases variability in observed data by creating error (E, F) around the underlying multidimensional distribution (D).

Three general forms of stochasticity influence community observations (or data, Fig. 1A): demographic stochasticity, environmental stochasticity, and measurement error (May 1973, Shaffer 1981, Lande et al. 2003). Demographic stochasticity describes the realized variability in intrinsic demographic processes (e.g., births, deaths, or migration) due to their probabilistic nature (Melbourne 2012). Here, different realizations of the same underlying demographic distribution create variability among individuals. In comparison, environmental stochasticity describes variability among realizations of extrinsic environmental conditions such as temperature, precipitation, and disturbances. Populations in a fixed environment can be described by a distribution of demographic traits, the variance of which is due to demographic stochasticity (Fig. 1B). When the environment is not fixed, environmental stochasticity creates variation in the probability that any given trait is expressed (Fig. 1C). These processes combine when considering demographic variation across variable environments (Fig. 1D). Imperfect data collection—often called measurement or observation error (Bolker 2008, Knape and de Valpine 2011)—can further cloud biological understanding, but does not interfere with ecological processes per se (Fig. 1E,1F). Our primary focus here is on the effects of demographic and environmental stochasticity on community dynamics and their predictability.

**Box 1. Illustration of our conceptual framework: How stochasticity affects individuals, populations, and communities**

**Figure 2:**
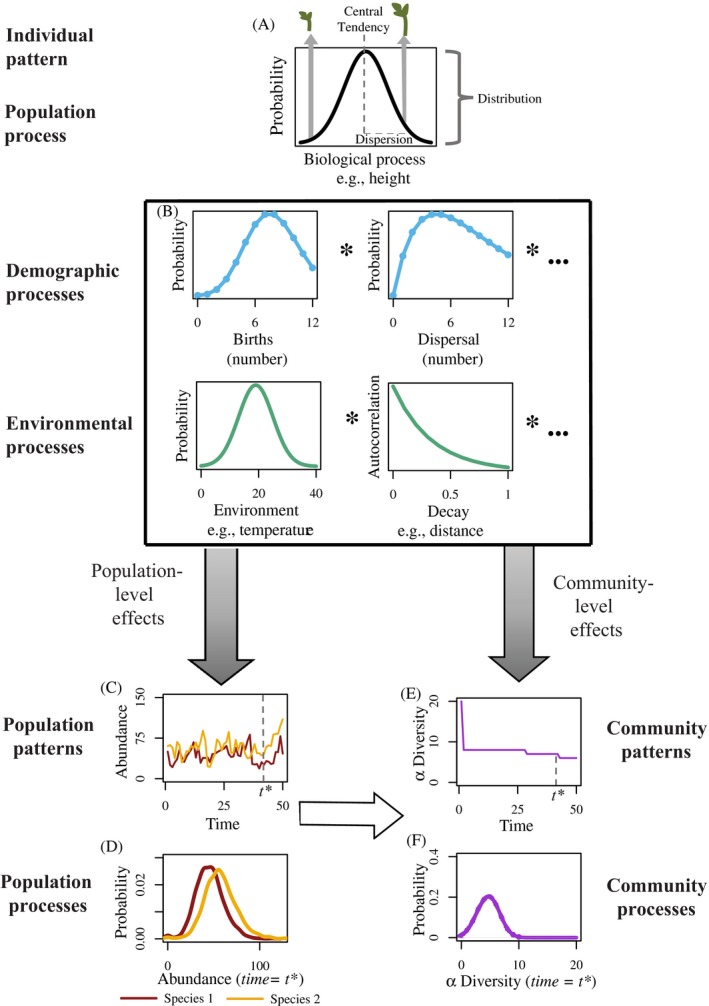
Visualization of our conceptual framework. Here we demonstrate the basic principles of our framework for examining the role of stochasticity in communities, as described in detail in Box 1. Direct applications of the framework occur in Figs. 3, 4, 5.

Although stochasticity is commonly associated with inherent uncertainty, stochastic processes are structured, as evidenced by their mathematical formalization. These processes can be described by distributions defined by parameters, including their central tendency (e.g., mean, median) and dispersion (e.g., variance; Fig. 2A). This simple principle holds true across levels of biological organization, where even population and community patterns, such as abundances and diversity, are defined by distributions of possible outcomes (Wu et al. 2006, Cabral et al. 2017).

Inherent variability in events described by stochastic processes is most evident when examining individual events. A single sampling event—for example, the height of a single seedling (Fig. 2A)—is random with respect to the underlying distribution (Vellend et al. 2014), in that it is impossible to predict the seedling’s height out of all biologically feasible possibilities. Once many seedlings from the population have been sampled, however, the underlying distribution can be described and it becomes possible to make a probabilistic prediction about the height of seedlings from this population process. Herein lies the structure of stochasticity. Although individual events are unpredictable, they can be described as “draws” from probability distributions and their outcomes can be predicted in the aggregate. Indeed, the distributions of stochastic outcomes might themselves be predicted from first‐principle predictions based on fundamental physical and chemical laws (West et al. 1999). Although this statement is eminently intuitive, we argue that its usefulness remains underexploited in ecology, where we tend to focus on comparing how mean patterns change across time or space (Chase et al. 2011, Vellend 2016), rather than how entire distributions deviate from one another.

Individuals, populations, and communities are influenced by a multitude of demographic and environmental processes (Fig. 2B). Therefore, it is essential to recognize that the patterns of sampled populations and communities represent the joint distribution of multiple demographic and environmental processes. Though this joint distribution may not be describable with a named distribution, it nonetheless exhibits measurable structure that can be estimated with enough draws from said distribution.

**Population and community outcomes of stochasticity**

Stochasticity propagates across levels of biological organization to affect population‐level patterns, such as the abundance of species through time (Fig. 2C). Even under constant abiotic and biotic conditions, we expect variability between draws (i.e., samples) of population events because of underlying stochastic processes. For example, an observed abundance pattern at a given time, t* (Fig. 2C), is the outcome of processes at the population level, which can be summarized by a distribution of likely outcomes representing the probability of the population’s abundance at time t* (Fig. 2D). Differences in abundance at time t* can arise in two ways: (1) as random draws from the same underlying population distribution of expected abundances, or (2) as draws from distinct distributions—representing different underlying assembly processes (Vellend 2016).

Despite the inherent variability associated with stochastic processes, the effects of stochasticity on ecological dynamics are often still predictable in a relative manner. An illustrative example arises from the classic work examining how variation in individual growth rate influences overall population growth (Ruel and Ayres 1999, Bolnick et al. 2011). With the nonlinear dynamics of Jensen’s Inequality (Jensen 1906), a population subject to density dependence with a distribution exhibiting smaller variance in individual growth rates will predictably have a larger population growth rate than a population with a larger variance in individual growth rates.

Once multiple populations begin interacting, stochastic effects propagate to alter community dynamics. Community‐level patterns, such as alpha diversity (Fig. 2E) can be both analogous to population‐level effects and propagate from populations to communities. Specifically, analogous effects of stochasticity on communities result from processes that are directly predictable from integrating several population‐level stochastic processes. This is similar to how individual‐level stochastic effects jointly generate stochasticity in populations. However, propagating effects of stochasticity on communities are considerably more complex, as they result from nonlinear interactions between multiple populations. This includes species interactions, such as competition (considered here), and could be easily extended to incorporate other interactions such as facilitation, food web dynamics, or spatial processes, which could lead to nonintuitive consequences for communities. As with population processes and patterns, observed community patterns, such as alpha diversity for a given community at time t*, are the result of draws from a community‐level distribution of possible alpha diversity values, yielding a predictable structure that emerges from stochasticity (Fig. 2F).

Our synthesis begins with a conceptual framework for better understanding how demographic and environmental stochasticity combine to provide predictable structure in diverse ecological communities (Box 1; Fig. 2). We anchor our framework with a modular population and community model that builds from classic models and incorporates both demographic and environmental stochasticity. This provides a flexible approach for (1) incorporating multiple sources of stochasticity in a single model and (2) connecting the rich literature of stochastic population ecology (reviewed in Boettiger 2018) and small assemblies of 2–3 species to diverse communities, where examples are far fewer. Focusing first on population persistence and community alpha diversity (local species richness), we use this common model to highlight multiple past modeling and empirical results and show that observed stochastic patterns in diverse communities are the result of the predictable nature of stochasticity. We then extend these insights beyond local coexistence and diversity patterns to metacommunities and diversity–stability relationships. Our goal is not to provide a comprehensive review of past results (for excellent recent reviews, see Vellend et al. 2014, Boettiger 2018), but rather to illustrate the utility of our approach for considering stochasticity as a structuring force in communities. After establishing the synthetic value of our framework, we offer guidance for future research that capitalizes on its lessons to improve our understanding of the role of stochasticity in diverse communities.

## Modeling Stochasticity in Populations and Communities

Our conceptual framework of the predictable effects of demographic and environmental stochasticity (Box 1) maps onto multiple types of models that incorporate stochasticity in various ways. Here we incorporate a stochastic version of the classic and well‐studied Beverton–Holt model (Beverton and Holt 1957) from population dynamics of a single species to diverse communities (in our examples, 20 species). We focus on this model for our synthesis because of its long history in both population and community ecology, its ability to be generalized to include other classic models (Brännström and Sumpter 2005), and its extensions that incorporate seed banks and stabilizing mechanisms of coexistence (Levine and HilleRisLambers 2009). Use of this model allows a concrete exposition of how the structure of stochasticity fundamentally changes community dynamics, while maintaining direct ties to the population theory from which we wish to build.

We begin with the deterministic Beverton–Holt population model, where

(1) $$N_{t+1}=RN_{t}\frac{1}{1+\alpha N_{t}}.$$

Here $N_{t}$ is the population size at time *t*, *R* is the density‐independent growth rate, and α is the intraspecific competition coefficient.

We incorporate demographic stochasticity in birth and mortality by drawing the population size in each time step from a Poisson distribution, where the expected population size is given by the deterministic Beverton–Holt model (Eq. 1; Shoemaker and Melbourne 2016):

(2) $$N_{t+1}∼\;\text{Poisson}\left(RN_{t}\frac{1}{1+\alpha N_{t}}\right).$$

We incorporate environmental stochasticity into Eq. 1 paralleling the classic work of Ripa and Lundberg (1996). Here environmental stochasticity σ is a temporally autocorrelated random variable. In this case

(3) $$N_{t+1}=RN_{t}\frac{1}{1+\alpha N_{t}}+N_{t}\;\zeta \;\sigma_{t}$$

where ζ controls the magnitude of the effect of environmental stochasticity on population dynamics at each time interval. The time series of environmental stochasticity to which a species responds is defined such that $\sigma_{t}=a\sigma_{t-1}+b\phi_{t}$, where $\sigma_{0}=0$ and b scales the magnitude of noise $\phi_{t}∼\text{Normal}\left(0,1\right)$. We set $b={\left(1-a^{2}\right)}^{0.5}$, which yields the convenient property that $var\left(\sigma \right)$ is the same for all a values (Ripa and Lundberg 1996). Environmental stochasticity can have a positively (0<a≤1), negatively (-1≤a<0), or uncorrelated (a=0) structure (Appendix S1).

Combining demographic and environmental stochasticity in populations yields

(4) $$N_{t+1}∼\text{Poisson}\left(RN_{t}\frac{1}{1+\alpha N_{t}}+N_{t}\;\zeta \;\sigma_{t}\right).$$

From the single‐species Beverton–Holt model, we then extend from populations to the community level (Shoemaker and Melbourne 2016), adding interspecific competition to Eq. 1 such that for each species *i*

(5) $$N_{i,t+1}=R_{i}N_{i,t}\frac{1}{1+\sum_{j}\alpha_{\mathrm{ij}}N_{j,t}}$$

where α*_ij_* is the pairwise competition coefficient of species *j* on species *i* (intraspecific competition is then considered when *j* = *i*). We use 20 species in our examples, but note that this model is general to any number of species.

Paralleling the methods used to add stochasticity to the population model, we add demographic stochasticity:

(6) $$N_{i,t+1}∼\text{Poisson}\left(R_{i}N_{i,t}\frac{1}{1+\sum_{j}\alpha_{\mathrm{ij}}N_{j,t}}\right),$$

environmental stochasticity:

(7) $$N_{i,t+1}=R_{i}N_{i,t}\frac{1}{1+\sum_{j}\alpha_{\mathrm{ij}}N_{j,t}}+N_{i,t}\zeta_{i}\sigma_{i,t},$$

or both:

(8) $$N_{i,t+1}∼\text{Poisson}\left(R_{i}N_{i,t}\frac{1}{1+\sum_{j}\alpha_{\mathrm{ij}}N_{j,t}}+N_{i,t}\zeta_{i}\sigma_{i,t}\right).$$

An additional source of demographic stochasticity may arise in communities via a distribution of interaction strengths between species pairs. For our examples, we consider α*_ij_* to be constant through time. However, we note that models that allow interaction networks to rewire (Kondoh 2003, Valdovinos et al. 2010, Nuwagaba et al. 2015) are an interesting avenue of future research. The full Eq. 8 provides a general framework and allows us to examine explicitly the effects of both demographic and environmental stochasticity as drivers of dynamics in diverse communities (Box 1).

Lastly, to demonstrate the versatility of our general approach and to incorporate additional biological realism via a seed bank—one of the classic examples where the storage effect may emerge (Chesson 2000)—we consider a stochastic model of a seed‐banking annual plant with Beverton–Holt dynamics (Levine and HilleRisLambers 2009),

(9) $$N_{i,t+1}=N_{i,t}s_{i}\left(1-g_{i,t}\right)+g_{i,t}R_{i}N_{i,t}\frac{1}{1+\sum_{j}\alpha_{\mathrm{ij}}N_{j,t}}.$$

Here $N_{i,t}$ is the number of seeds of species *i* at the beginning of growing season *t*, $s_{i}$ is the survival of ungerminated seeds in the seed bank, and $g_{i,t}$ is the fraction of seeds that germinate in a given year. In the deterministic version of the model, $g_{i,t}=g_{i}$, i.e. is constant in time. To incorporate autocorrelated environmental stochasticity, we use $g_{i,t}=g_{i}+\zeta_{i}\sigma_{i,t}$ (although restricted to be between 0 and 1), with ζ*_i_* and σ*_i,t_* as in Eq. 3. In all models, extinction events are defined when population size drops below one individual. Model code is archived on Zenodo,1
https://zenodo.org/record/3455859#.XYVKtpNKj_Q
 and an overview of all models is given in Appendix S3.

**Figure 3:**
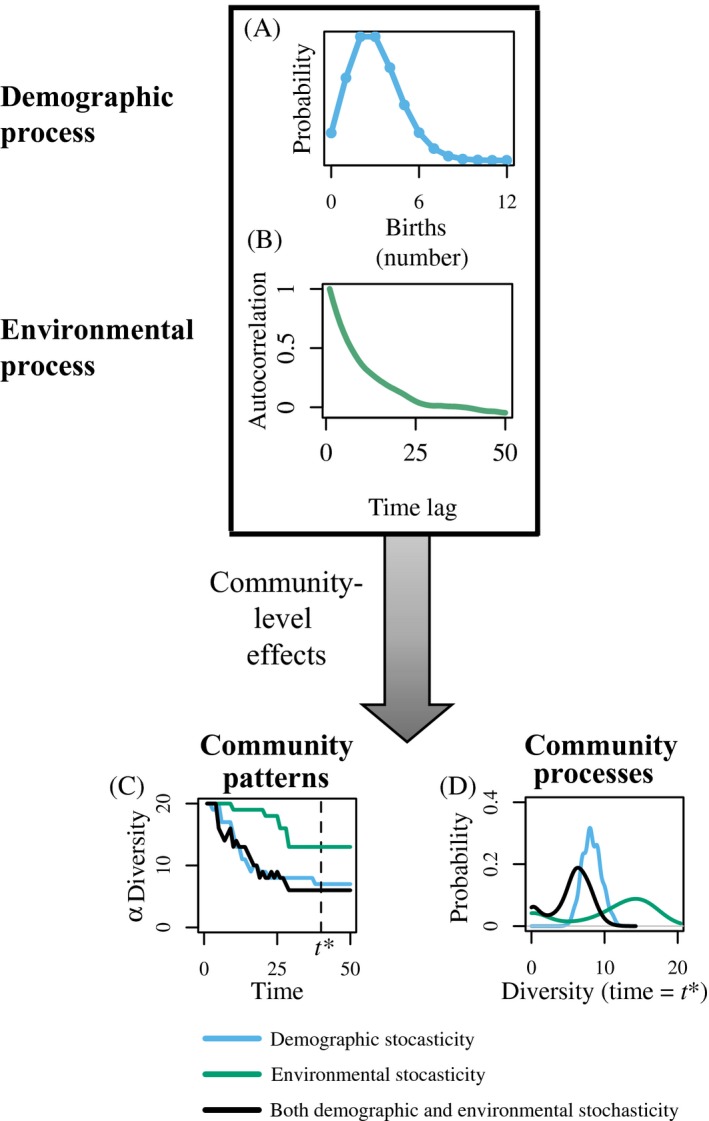
Incorporating both demographic and environmental stochasticity concurrently. Even when incorporating relatively simple demographic (A) and environmental (B) stochastic processes, observed community patterns (C) and their underlying distributions (D) differ when considering only a single type of stochasticity rather than their combination. Model parameters are a=0 (no autocorrelation), ζ=0.35, $R_{i}∼\text{Uniform}\left(2,2.5\right)$ $\alpha_{\mathrm{ij}}∼\text{Uniform}(0.005,0.01$) when i≠j, and $\alpha_{\mathrm{ii}}=0.03$ for all *i*, *j* (Eqs. (5), (6), (7), (8)). Distributions of expected diversity are created at time point *t = *40 by examining observed diversity across 1,000 runs.

Our modeling framework provides a straightforward demonstration of how omitting all (Eq. 5) or some (Eqs. 6 and 7) forms of stochasticity from an analysis yields alternative predictions to when both demographic and environmental stochasticity act concurrently (Eq. 8). For example, models yield different predictions for alpha diversity dynamics through time depending on whether they include demographic (Fig. 3A, Eq. 6), environmental (Fig. 3B, Eq. 7), or both forms of stochasticity (Fig. 3C, D, Eq. 8). Although differences in any one set of observations may appear subtle at times (Fig. 3C), the underlying distributions of predicted outcomes fundamentally differ in both their mean and variance (Fig. 3D).

## Examining Past Results Through a Common Modeling Framework

As even subtle shifts in stochastic patterns have the potential to transform population and community characteristics (Williams and Hastings 2011), identifying general rules for how stochasticity influences patterns of diversity in communities has been a key challenge in both theoretical and empirical community ecology. To synthesize previous work in a single framework (Box 1), we use our model to illustrate well‐developed insights from the stochastic population ecology literature (e.g., single species models, simple two‐species competitive or predator–prey models; see also Boettiger 2018) and the burgeoning work in higher‐diversity communities (Ruokolainen and Fowler 2008, Miller et al. 2011, Ai et al. 2012, Vellend et al. 2014, Gilbert and Levine 2017). We focus on persistence at the population level (May 1974, Morris and Doak 2002, Drake 2006) and extend these observations to species richness at the community level (Chesson 2000, Vellend 2016). By uniting previous studies in a single framework, we show how both demographic and environmental stochasticity can produce predicable dynamics when scaling from populations to communities.

### Demographic stochasticity

**Figure 4:**
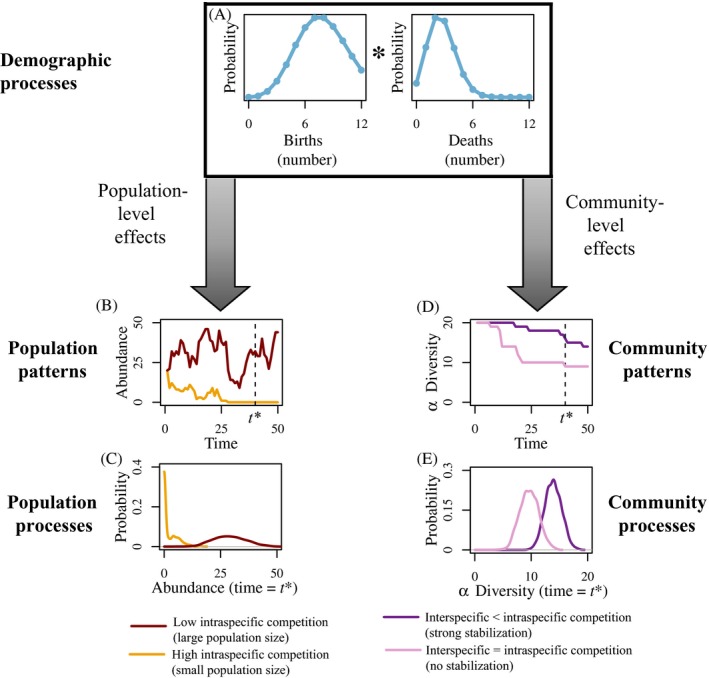
Modeling demographic stochasticity in populations and communities. Modeled effects of demographic stochasticity (A) alter both population abundance and community diversity patterns. In accordance with the literature, we focus on the interaction of demographic stochasticity with carrying capacity in populations and intra‐ and interspecific competition in communities. At the population level, persistence time declines with increasing demographic stochasticity in small populations (B), as the distribution of expected species abundances decreases with decreasing population size (C). At the community level, observed alpha diversity (D) is expected to be more variable and decline faster over time when intraspecific competition ≈ interspecific competition than when the strength of interspecific competition is less than that of intraspecific competition (E). Population model parameters are $R_{1}=R_{2}=1.6$, $\alpha_{1}=0.02$, $\alpha_{2}=0.1$ (Eq. 2). Community model parameters are (1) purple line $R_{i}∼\;\text{Uniform}\left(2.0,2.5\right)$ $\alpha_{\mathrm{ij}}∼\text{Uniform}\left(0.002,0.005\right)$ when i≠j, and $\alpha_{\mathrm{ii}}=0.03$ and (2) pink line $R_{i}=\bar{R}$ from the purple line and $\alpha_{\mathrm{ij}}=\bar{\alpha_{\mathrm{ij}}}$ from the purple line for all *i*, *j* (Eq. 6) where the overbar denotes the mean.

Demographic stochasticity produces fluctuations in population abundance through time via underlying probability distributions of demographic events (i.e., births and deaths; Fig. 4A; Eq. 2), which strongly influence population persistence and their probability of extinction (Lande 1993). Effects on persistence are particularly relevant in populations with low carrying capacities (i.e., strong intraspecific competition in Eq. 2), and decrease as population carrying capacity increases (as in Fig. 4B; comparing yellow vs. red time series; MacArthur and Wilson 1967, Gabriel and Bürger 1992, Lande 1993). Indeed, as population carrying capacity increases, it is well established that mean time to extinction increases exponentially because of decreasing effects of demographic stochasticity (Fig. 4C; Ovaskainen and Meerson 2010, Melbourne 2012). In small populations, few demographic events can have a large effect if they are taken from the extremes of the distribution (Caughley 1994, Hedrick et al. 1996), and thus populations may trend toward extinction with each unfortunate dip in population growth (Fig. 4B). The result is a predictably high probability of extinction for small populations at relatively short time scales and a skewed distribution of abundances (Fig. 4C). In comparison, in larger populations observed demographic distributions will closely match the mathematical expectation, thereby buffering populations from extinction from demographic stochasticity.

Empirical systems corroborate that stochastic demographic structure can yield predictable outcomes. For instance, a study of 359 populations of eight threatened species in Northern Germany found that, over 10 yr, small populations were more likely to go extinct than larger ones (Matthies et al. 2004). Critically, not all small populations went extinct. Thus, although it is not possible to predict single stochastic events, such as when and whether a particular population may go extinct, overall we see consistent and predictable outcomes across populations. More generally, this predictable outcome of demographic stochasticity has greatly influenced the field of conservation biology, where the International Union for Conservation of Nature (IUCN) uses population size (and correspondingly how prone a species is to “stochastic events”) as one benchmark in assessing species’ vulnerability to extinction (IUCN 2017).

Demographic stochasticity can also influence diversity in communities with stochastic species‐interaction strengths and niche differentiation (Adler and Drake 2008, Pedruski et al. 2015), and our framework illustrates when and why this occurs (extending from Eqs. 2 to 6). When local communities exhibit strong stabilizing niche differences, we find that alpha diversity patterns tend to be largely unaffected by demographic stochasticity (dark purple curve in Fig. 4D, E). Here, strong niche differences overwhelm the effects of stochastic demographic rates, consistent with theoretical expectations (Orrock and Watling 2010, Ai et al. 2012). The role of demographic stochasticity is minimal, and other forms of variability (e.g., environmental heterogeneity, temporal seed banks) strongly stabilize community diversity (Chesson 2000). Thus, we observe that the distribution of predicted alpha diversity in these communities has relatively low variance (dark purple curve in Fig. 4E; Zillio and Condit 2007).

The impacts of stochasticity on alpha diversity differ predictably from above when interactions among equivalent species drive community dynamics (e.g., neutral theory; Hubbell 2001). Here, community patterns like alpha diversity become more variable when assuming neutral dynamics (pink curve in Fig. 4D, E). Predictably, greater variance in alpha diversity occurs in neutral compared to nonneutral communities (pink curve vs. purple curve in Fig. 4E) and diversity patterns appear to drift through time (Vellend et al. 2014, Vellend 2016).

### Environmental Stochasticity

**Figure 5:**
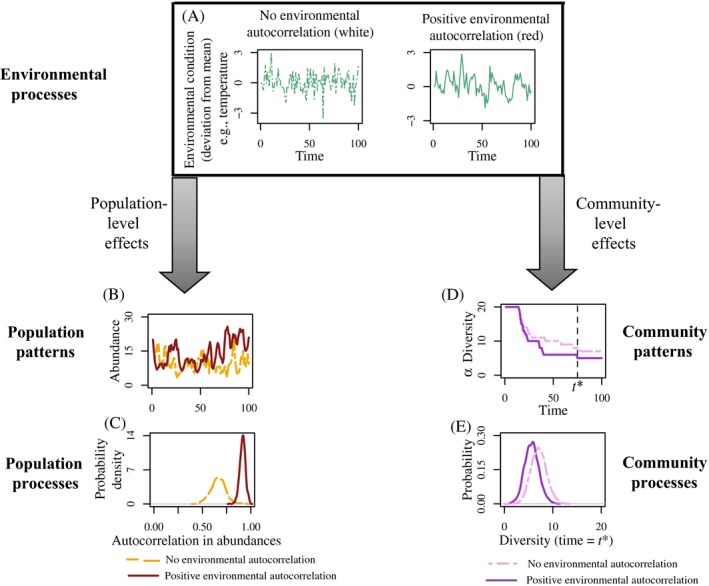
Modeling environmental stochasticity in populations and communities. Modeled effects of autocorrelation in environmental stochasticity (A) alters population and community patterns (a=0 for white noise; a=0.75 for red noise). In compensatory populations, increasing the strength of positive autocorrelation in environmental stochasticity increases the correlation in population size between time steps (B, C). In contrast, in communities where environmental stochasticity alters the germination rate from the seedbank, environmental autocorrelation has a more minimal effect, but increasing the strength of positive autocorrelation in environmental stochasticity slightly decreases expected diversity (D, E). Population model parameters are R=1.5,ζ=0.25, and α=0.05 (Eq. 3). Community model parameters are $s_{i}=0.8,\;g_{i}=0.5,\;\zeta =0.2,R_{i}∼\;\text{Uniform}\left(1.1,1.5\right)$ and $\alpha_{\mathrm{ij}}∼\text{Uniform}\left(0.001,0.005\right)$ when i≠j, and $\alpha_{\mathrm{ii}}=0.007$ (Eq. 7).

Environmental stochasticity affects population persistence through extrinsically‐derived processes (Fig. 5A, Eq. 3; Lande 1993). In particular, the autocorrelation of environmental conditions strongly influences population persistence (Ripa and Heino 1999, Fagan et al. 2001, Engen et al. 2002, Benton et al. 2004, Drake and Lodge 2004, Schwager et al. 2006, Ruokolainen and Fowler 2008). In our model, we compare white (no autocorrelation) and red (positively autocorrelated) noise (Fig. 5A), as this range is most relevant in ecological settings (Vasseur and Yodzis 2004).

Predictable outcomes for population persistence depend on the interplay between population density dependence (i.e., compensatory dynamics) and the structure of the autocorrelation in environmental stochasticity (Ruokolainen et al. 2009). In the case that population dynamics are *compensatory*, as in the Beverton–Holt model, population dynamics closely track the sign of autocorrelation in environmental condition. While correlations between population size at time *t* and time *t* + 1 are expected regardless of environmental stochasticity, increasing the autocorrelation of environmental stochasticity increases the correlation of population dynamics (Fig. 5B, C; Eq. 3), as predicted by Kaitala et al. (1997).

However, when populations exhibit *overcompensatory* dynamics (i.e., growth rates respond more strongly than is needed to compensate for changes in environmental condition, causing an over‐ or undershoot of the carrying capacity and often oscillatory dynamics), persistence time predictably increases as the strength of positive autocorrelation increases (Petchey et al. 1997, Ripa and Heino 1999); our modeling framework also reproduces this effect if we replace Beverton–Holt density dependence with a form that allows overcompensation, such as the Ricker model (Appendices S3 and S4). Intuitively, this occurs because increasing positive environmental autocorrelation dampens overcompensatory population fluctuations. This decreases the risk that a species will go extinct while at high abundance (Ripa and Heino 1999). Empirical observations testing this theory corroborate these predictions (Widarto et al. 2007, Colchero et al. 2009). For example, experimentally imposing increasingly positive autocorrelation in environmental stochasticity increased population persistence in the arthropod *Folsomia candida* (Pike et al. 2004).

In *undercompensatory* populations (i.e., populations where growth rates are smaller than necessary to compensate for the decreased population size following an environmental perturbation), persistence tends to decrease with increasing positive autocorrelation in environmental stochasticity compared to environments with no autocorrelation (Petchey et al. 1997, Cuddington and Yodzis 1999). However, conflicting predictions arise (Petchey et al. 1997, Heino et al. 2000) because persistence time in undercompensatory populations depend strongly on the effects of either a single catastrophic event, or a series of poor environmental conditions (i.e., ‘pulse’ and ‘press’ events, respectively). Positive autocorrelation in environmental conditions decreases the risk of an isolated catastrophic event, but increases the likelihood of a large time span (for temporal autocorrelation) or area (for spatial autocorrelation) of poor environmental conditions, rendering it difficult to predict how environmental stochasticity will affect the persistence of undercompensatory populations (Schwager et al. 2006).

Studies of population vs. community dynamics have historically taken different approaches to characterizing the structure of environmental stochasticity, yet both demonstrate its predictable nature. Although most population‐level studies describe stochastic events in terms of variance and autocorrelation, many community studies instead emphasize the frequency and intensity of these events (Miller et al. 2011). In communities, altering the frequency or intensity of stochastic events can increase environmental variation and promote the maintenance of biodiversity in communities (Chesson 2000, Adler and Drake 2008).

Temporal and spatial variation in the environment, such as through environmental stochasticity, can predictably allow for coexistence when species benefit from different environmental conditions via niche partitioning (Chesson 2000, Levine and Rees 2004, Usinowicz et al. 2012). If species can “store” these benefits from favorable years or locations through poor conditions (e.g., through a seed bank or long‐lived adults), then environmental variability can promote coexistence via the storage effect (Chesson 2000). Incorporating autocorrelated environmental variation that alters germination from a seed bank into our model, we find that increasing positive autocorrelation drives a decrease in alpha diversity compared to uncorrelated environmental perturbations (Fig. 5D, E; Eq. 9). Our result matches those presented by Ruokolainen and Fowler (2008), where increasing positive autocorrelation of environmental stochasticity increases extinction risk when species respond to different underlying environmental drivers (Ruokolainen and Fowler 2008). This occurs because increasing the autocorrelation of environmental stochasticity increases variance in population abundances, making extinction events more likely. Future work is necessary to verify these results across model assumptions and to explore more complex nuances, such as having environmental stochasticity explicitly alter the covariance between species’ traits (such as competitive ability and germination rates) or exploring the relationship between autocorrelation of environmental stochasticity and other fluctuation‐dependent coexistence mechanisms (e.g., relative nonlinearity in growth responses and growth‐density covariances; Snyder and Chesson 2004, Snyder 2008).

## Extensions to Metacommunities and Stability Relationships

The importance of stochasticity for community dynamics extends beyond local coexistence and diversity patterns. Notably, both demographic and environmental stochasticity are integral in extending metapopulation theory (Hanski and Gilpin 1997, Gilpin 2012) to incorporate spatial community dynamics (Tilman 1982, 1994, Holyoak et al. 2005, Leibold and Chase 2018). Central to metacommunity theory is the interplay between demographic rates such as dispersal (Büchi and Vuilleumier 2014, Lowe and McPeek 2014) and spatial variability arising from environmental stochasticity. For example, the stochastic nature of dispersal inherently increases the use of sink habitat, thus creating an opportunity for source–sink metacommunity dynamics (Pulliam 1988), regardless of dispersal strategy (passive, density‐dependent, or based on behavioral decisions; Amarasekare 2006, Patten and Kelly 2010, Lowe and McPeek 2014). Here, an interesting interaction between demographic and environmental stochasticity naturally arises: high variability in dispersal kernels plays a key role in creating sink populations as individuals have a nonzero chance of dispersing to unsuitable habitat, while positively autocorrelated environmental stochasticity inflates local abundances in sink populations, thus increasing time to extinction (Gonzalez and Holt 2002, Roy et al. 2005). More generally, many foundational metacommunity frameworks (e.g., patch, source–sink and species sorting dynamics) incorporate spatiotemporal environmental variability (Tilman 1994, Tilman et al. 1994, Mouquet and Loreau 2003). This variability is often invoked to represent demographic (e.g., random mortality events) or environmental (e.g., disturbance) stochasticity. However, many of these models rely fully on deterministic mean‐field approximations, rather than incorporating probabilistic distributions of stochastic events. Future work incorporating distributions of predicted outcomes (Box 1) will allow community ecologists to mechanistically parse out the importance of stochasticity vs. other sources of variability for metacommunity dynamics.

The interplay of stochastic mechanisms and species diversity also has profound effects on community stability, such as the coefficient of variation of aggregate biomass or abundance (Donohue et al. 2013, Loreau and de Mazancourt 2013). Diversity–stability relationships cannot be understood outside the context of their drivers (Ives and Carpenter 2007), which themselves may be stochastic by nature (Yang et al. 2019). Even with highly stochastic environmental fluctuations, biodiversity can stabilize community dynamics more than would be expected in single populations, as statistical averaging may yield an aggregate community that is less volatile (e.g., with less variation in biomass) than each individual species (Doak et al. 1998, Schindler et al. 2015). Species may also show complementary responses to stochastic drivers via variation‐dependent coexistence mechanisms and niche partitioning (Chesson 2000, Barabás et al. 2018), which increases stability of community diversity compared to population‐level abundance patterns (Loreau et al. 2003). Here, demographic stochasticity can increase variability in abundances among species, which can either help to stabilize community dynamics by decreasing synchrony between species (Gouhier et al. 2010, Loreau and de Mazancourt 2013), or can destabilize communities by amplifying species’ extinction risks at low population sizes (Tilman et al. 1998, Dennis et al. 2016). Again, considering the underlying autocorrelation in and distributions of drivers represents a critical step forward. For example, in the model formulation of Yang et al. (2019), the temporal autocorrelation of environmental factors is more important in driving community stability than underlying food‐web characteristics such as number and type of species interactions.

## Insights and Future Directions

Knowledge of stochastic structure is fundamental to improving our understanding of—and our ability to predict—the dynamics of communities (Vellend 2010, 2016). Our model demonstrates that both demographic and environmental stochasticity can significantly alter diversity dynamics and their underlying distributions in species‐rich communities (Fig. 3; Eq. 8), which likely have broad implications at higher levels of biological organization (de Mazancourt et al. 2013). We advocate for future work that focuses on the simultaneous investigation of both demographic and environmental stochasticity and their underlying distributions to advance understanding of community dynamics in an inherently stochastic world.

**Figure 6:**
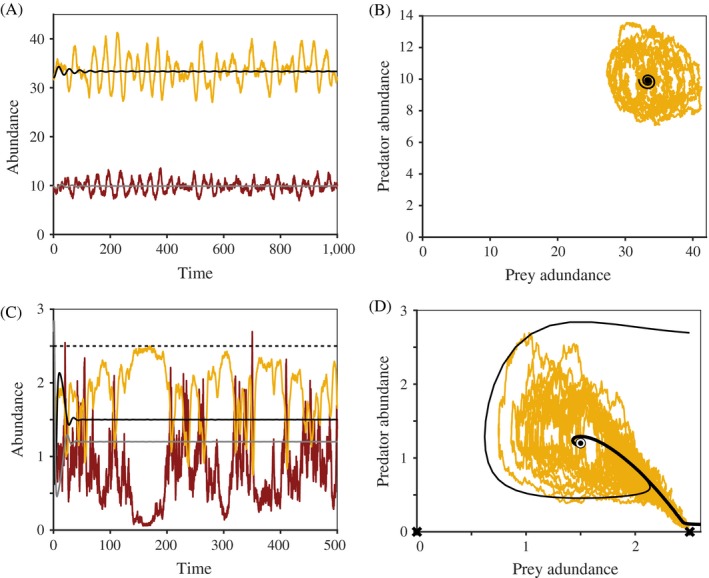
The role of stochasticity in transient dynamics. Predator–prey dynamics, plotted both as time series (left) and as phase diagrams (right), with stochastic trajectories shown in yellow or red and deterministic trajectories in black or gray. (A) The deterministic model (deterministic prey dynamics in black and deterministic predator dynamics in gray) shows transient cycles. In the presence of environmental stochasticity (stochastic prey dynamics in yellow and stochastic predator dynamics in red), the cycles are sustained. Here, stochasticity prevents the populations from settling onto their deterministic equilibrium and the cycles that were transient in the deterministic case are perpetuated forever. (B) The same dynamics in state space, with a stochastic trajectory in yellow and a deterministic one in black. (C) Another stochastic predator–prey model, illustrating how stochasticity can reveal unstable features in the underlying deterministic structure. Without stochasticity (black and gray lines), the populations show transient cycles then settle onto a stable coexistence equilibrium. An unstable equilibrium exists at the dashed black line (for the prey) and the *x*‐axis (for the predator). With stochasticity (red and yellow trajectories), the populations visit both the stable and unstable equilibria. Thus, the stochastic dynamics can reveal the unstable states in a system. (D) shows these dynamics in state space, with the deterministic equilibria marked: the dot is the stable equilibrium and Xs are unstable (saddle) points. Models are described in Appendices S3 and S5.

Future theoretical insights for community ecology will come from continued incorporation of stochasticity into deterministic modeling frameworks (Ripa and Lundberg 1996, Petchey et al. 1997). For example, community‐level analytical models (Melbourne and Hastings 2008), computational simulations (Katul et al. 2005), and individual‐based models (Wiegand et al. 2004, Taubert et al. 2012) highlight how to incorporate different types of stochasticity into a deterministic model skeleton, thereby isolating and enabling quantitative comparison of the stochastic effects of both demographic and environmental stochasticity. As a classic population example, Lande (1993) follows this methodology, showing that for large local populations, environmental stochasticity dwarfs the effects of demographic stochasticity in causing extinctions. We encourage theoreticians to continue these explorations by either (1) building on classic stochastic population models to consider a greater number of species, or (2) incorporating stochasticity into existing multispecies frameworks (Allesina and Levine 2011, Saavedra et al. 2017). In addition, although the majority of the ecological literature focuses on the role of stochasticity in equilibrium dynamics, stochasticity also elucidates underlying deterministic processes in transient systems (Schaffer et al. 1986, Stouffer et al. 2018)—an exciting avenue for future exploration (Box 2; Appendix S5; Fig. 6).

**Box 2. Deterministic factors also have structure, and stochasticity can help reveal it**

Population and community dynamics are often studied in the context of an equilibrium—the long‐term theoretical expectation for how a system behaves in the absence of stochasticity. Stochasticity, whether demographic or environmental, can prevent populations and communities from ever settling permanently onto their theoretical equilibrium. In this way, stochasticity reveals a population or community’s *transient dynamics*—its behaviors when not at equilibrium.

Transient dynamics constitute a predictable response to stochastic perturbations because, in addition to being shaped by structure in the stochasticity itself, transients emerge from structure in deterministic processes (Higgins et al. 1997, Hastings 2004, Hastings 2010, Hastings et al. 2018). For example, the deterministic negative feedback inherent in consumer‐resource interactions makes these systems prone to cycling (Murdoch et al. 2003). When the cycles are transient, systems will theoretically settle onto a point equilibrium in the absence of stochasticity (Fig. 6A, black and gray trajectories; Fig. 6B, black trajectory). With stochasticity, the same systems would exhibit sustained cycles (Fig. 6A, yellow and red trajectories; Fig. 6B, yellow trajectory). After the unperturbed system equilibrates, the underlying propensity to cycle is entirely hidden, even though the feedback responsible for cycles is deterministic. That stochasticity can reveal additional consequences of density‐dependent species interactions and feedbacks, beyond just their stable equilibria, is a powerful observation with two implications that we explore below.

1. The exciting implication: a fuller understanding of deterministic structure should help us interpret and predict some effects of stochasticity.

Our understanding of stochastic population dynamics is much improved if we acknowledge that unstable portions of the deterministic structure (e.g., saddles, unstable cycles as in Fig. 6A, B, and unstable chaos) are sometimes just as important as the stable portions. For example, Cushing et al. (1998) concluded that what appeared to be alternative stable states in laboratory populations of *Tribolium* beetles was actually a single stable state and a saddle (an unstable equilibrium that is approached transiently from some directions) revealed by stochasticity. Abbott and Nolting (2017) found a similar result in a stochastic predator–prey model (Fig. 6C, D). Even unstable chaotic oscillations can dominate stochastic dynamics (Kendall 2001, Dwyer et al. 2004). In population ecology, this important lesson can be readily applied because there are established methods for finding both stable and unstable equilibria in population models.

The applicability of this lesson to community ecology is not well understood, likely for several reasons. First, many quantities of interest in communities (e.g., evenness, beta diversity) are computed a posteriori from data or simulation results, not calculated directly from demographic and interaction rates. For quantities like these, there is no clear notion of what an equilibrium value is, much less an unstable equilibrium. Second, even if we examine community composition in terms of species abundances, to have a clear equilibrium concept to apply, equilibria of many‐species systems usually cannot be found analytically. Stable equilibria can be found through simulation, but unstable equilibria are much more challenging to identify (Sieber et al. 2013). By extension, it is much harder to look for any influence of unstable equilibria in communities. Here, population ecology grants us what could be a valuable idea: when stochastic community dynamics look quite different from any known stable states, it may be productive to search for an unstable community state in the vicinity of the stochastic dynamics. The possibility that unexplained patterns in communities may be driven by unstable structures revealed by stochasticity is an exciting prospect.

(2) The daunting implication: we may not be able to tell from observed dynamics whether a population or community is at equilibrium, or whether stochasticity is keeping it in its transient phase.

The processes and parameters that most strongly shape population dynamics (Chen and Cohen 2001)—population persistence (Hastings 2001), community assembly (Hein and Gillooly 2011), and alternative states (Fukami and Nakajima 2011, 2013)—can differ greatly depending on whether a system is at equilibrium or undergoing transient dynamics. Highly stochastic communities may recapitulate transient processes like community assembly indefinitely, whereas systems not as strongly influenced by stochasticity may converge on communities at a stable equilibrium. Because stochasticity can reveal both equilibrial and nonequilibrial behaviors, we may not be able to tell whether a particular community is in or out of equilibrium, and inferring process from observed patterns is likely to be problematic (Hastings et al. 2018). Efforts to integrate transient and equilibrial theory into community ecology (Fukami 2015, Stouffer et al. 2018) and to extend nonequilibrial tools from population to community ecology (Barabás et al. 2014) are likely to be particularly fruitful for understanding stochastically perturbed communities.

Experimental work also has great potential to explore the role of stochasticity in driving community dynamics. For example, microcosm studies have made significant advances in exploring stochasticity and connecting experiments to theory (Benton et al. 2001, 2004, Drake and Lodge 2004, Reuman et al. 2006), as they have the advantage of relatively low measurement error, highly controlled conditions, and high replication. This type of experimental setup elucidates the underlying probability distributions for both demographic and environmental processes. For example, Benton et al. (2004) were able to manipulate the importance of demographic stochasticity by experimentally perturbing vital rates and turning environmental stochasticity on and off via either a constant or a variable rate of food availability in a soil mite (*Sancassania berlesei*) system. We suggest these methods can be extended for use in traditional community microcosm systems and can be augmented with increased diversity to couple theory and empirical tests exploring the role of stochasticity in communities.

An exciting approach for understanding stochastic effects on communities is to manipulate the effects of stochasticity directly in the field in order to isolate and quantify the effect of a given stochastic process (Gilbert and Levine 2017). This type of manipulation is becoming increasingly common, with more researchers describing demographic distributions via experiments (Levine and HilleRisLambers 2009, Sullivan et al. 2018), and manipulating demographic variability (Germain et al. 2017) or environmental condition (Hawkes et al. 2011, Liu et al. 2015). We suggest next steps include manipulating both types of stochasticity in communities in a factorial fashion, allowing for close integration of stochastic theoretical advances with experiments in diverse communities. Experiments that manipulate population size provide information on the importance of demographic stochasticity, because larger populations experience less of an influence on demographic stochasticity (Gilbert and Levine 2017). Density manipulations could then be crossed with environmental manipulations to determine how demographic and environmental stochasticity alter communities independently and concurrently. Combining these types of experiments with concurrent theoretical models that inform the manipulations of stochastic parameters would provide additional insight.

Because they complement experimental approaches to tease out stochasticity directly, appropriate statistical methods can assist empiricists attempting to address questions related to stochasticity in community data. Methods adapted from population biology can be used to measure the stochastic signature in community data when time series exist for multiple species. For example, multivariate autoregressive models, such as those developed by Ives et al. (2003), can tease apart environmental stochastic effects and observation error from time‐series data of multiple species in a community through the inclusion of covariance structures. Null model methods have also been developed to determine the relative importance of stochasticity in highly diverse communities (Chase et al. 2011, Kraft et al. 2011). However, these methods are not ideal for inferring underlying process from patterns (Gotelli and Ulrich 2012, Tucker et al. 2016), in large part because they often assume that stochasticity merely adds increased variability to communities and fail to recognize the inherent structure created by stochasticity. Spatial point pattern analysis is another avenue for quantifying species patterns in fully mapped census plots (Wiegand et al. 2017). When spatial pattern data are compared with simulation models (Grimm et al. 2005, Hartig et al. 2011), alternative hypotheses on spatial processes and drivers of stochasticity can be generated (May et al. 2015, 2016). Bayesian approaches also complement stochastic community ecology by allowing for analyses that use empirically based distributions of traits instead of averaged point estimates or assumed distributions (Clark 2010). Bayesian methods can be advantageous, as they inherently account for empirical data resulting from the combined effects of multiple demographic and environmental stochastic processes (as well as measurement error), and thus can capture stochastic structure that may not follow named distributional patterns. Because at times measurement error yields high variability (Crone 2016; Fig. 1), we encourage empiricists to estimate measurement error directly (e.g., determining the probability of refinding tags or other markers that are known to exist) or use methods that improve precision and accuracy of data collection (e.g., mark–recapture models, rare occurrence estimation; Jolly 1982) to tease apart biologically relevant stochasticity from measurement error.

## Conclusion

Stochasticity is more than merely reflective of uncertainty; it has biological structure that leads to predictable outcomes for population and community dynamics. We demonstrate this using a common modeling approach where we consider both demographic and environmental stochasticity and their combined effect. Across ecological communities, the key processes that shape dynamics are by their very nature stochastic, and therefore understanding their underlying distributions is fundamental for predicting patterns in community data and connecting patterns to processes.

## Data Availability

Data are available from Zenodo: http://doi.org/10.5281/zenodo.3455859
