# Supplementary material for: Integrating the underlying structure of stochasticity into community ecology
Source: Ecology. 2019 Dec 26;101(2):e02922. doi: 10.1002/ecy.2922 (PMC7027466; doi:10.1002/ecy.2922)
Supplement: Supplementary file 1 [file ECY-101-e02922-s001.pdf]

Supporting Information. Lauren G. Shoemaker*, Lauren L. Sullivan*, Ian Donohue, Juliano S. Cabral, Ryan J. Williams, Margaret M. Mayfield, Jonathan M. Chase, Chengjin Chu, W. Stanley Harpole, Andreas Huth, Janneke HilleRisLambers, Aubrie R.M. James, Nathan J.B Kraft, Felix May, Ranjan Muthukrishnan, Sean Satterlee, Franziska Taubert, Xugao Wang, Thorsten Wiegand, Qiang Yang, Karen C. Abbott. 2019. Integrating the underlying structure of stochasticity into community ecology. *Ecology.*

* Denotes equal contribution and corresponding authors

Appendix S1: Glossary

Colors of noise: Defines the autocorrelation (spatial or temporal) of a series of stochastic events. Redder color means stronger positive autocorrelation where subsequent draws from a distribution are likely to be similar to the current draw. Bluer is stronger negative autocorrelation, where subsequent draws from a distribution are more likely to be different from the current draw. White noise has no autocorrelation.
Deterministic: A deterministic process is one with no variability or randomness, i.e. all realizations of the process are identical. A deterministic system would always exhibit the same behavior given precisely the same starting conditions. Chaos, in a mathematical sense, is an example of a deterministic process. While it can appear completely random and lacks a repeating pattern, it is in fact derived from deterministic processes. It is highly dependent on starting conditions, but could be re-created if they were known precisely.
Distribution: A probability density function (continuous variables) or probability mass function (discrete variables) that describes a stochastic biological or environmental process. These distributions can be defined by many parameters, such as central tendency (e.g. mean, median, mode) and dispersion (e.g. variance, skew). Each individual draw from a distribution is a random event. Given infinite draws, the quantity would be completely described by the distribution of these draws.
Error: Statistical uncertainty related to collected data. This uncertainty can arise from process error – underlying stochasticity in processes (e.g. births, deaths, dispersal, etc.), or measurement/ observation error – imperfect ability to accurately collect data.
Heterogeneity: Dissimilarity among multiple parts of a system (e.g. patches in a metacommunity). Heterogeneity can arise from either deterministic processes (e.g. patch 1 has different soil conditions than patch 2) or stochasticity processes (e.g. the moisture conditions of each patch are drawn from the same underlying stochastic distribution and result in different observed conditions).
Random: A lack of pattern or predictability of a single event. In a biological context, a random event refers to a single sample from any process (i.e. draw from any distribution) that can be described by a stochastic distribution.
Stochasticity: A process that can be represented in a probabilistic way defined by its parameters (e.g. mean, variance, and skew). For a given biological process, repeated sampling from the same underlying distribution results in inherent variation between observed individual random

outcomes. There are several mechanisms that produce stochasticity in ecological systems; they can be categorized as either demographic or environmental stochasticity, or as measurement error (see Fig. 1, Box 2).
Transient dynamics: The behavior of a system that is not at equilibrium. Transience can result from deterministic or stochastic perturbations to a system.
Variability: A general term used to represent a range of possible outcomes for a process or quantity that can arise from incomplete sampling of stochastic distributions. While stochasticity is often used synonymously with variability, variability comes from multiple sources. Stochasticity is one of those sources, but other sources include: heterogeneity, age structure, individual variation etc., which could be deterministic or stochastic.
