# Supplementary material for: Integrating the underlying structure of stochasticity into community ecology
Source: Ecology. 2019 Dec 26;101(2):e02922. doi: 10.1002/ecy.2922 (PMC7027466; doi:10.1002/ecy.2922)
Supplement: Supplementary file 2 [file ECY-101-e02922-s002.pdf]

Supporting Information. Lauren G. Shoemaker*, Lauren L. Sullivan*, Ian Donohue, Juliano S. Cabral, Ryan J. Williams, Margaret M. Mayfield, Jonathan M. Chase, Chengjin Chu, W. Stanley Harpole, Andreas Huth, Janneke HilleRisLambers, Aubrie R.M. James, Nathan J.B Kraft, Felix May, Ranjan Muthukrishnan, Sean Satterlee, Franziska Taubert, Xugao Wang, Thorsten Wiegand, Qiang Yang, Karen C. Abbott. 2019. Integrating the underlying structure of stochasticity into community ecology. *Ecology*.

* Denotes equal contribution and corresponding authors

Appendix S2: Stochasticity Word Association Methods and Results

Semantics of Stochasticity: Syntax and Context

The term "stochasticity" has been used in a multitude of ways in ecology (Vellend *et al.*, 2014) with interpretations being largely context-dependent. This has resulted in similar language being used to refer to different phenomena, which has led in turn, to a general confusion about stochasticity and its value for understanding ecological processes. To demonstrate this, we use a word association approach to search the literature for correlations between words related to stochasticity and examine how this language is used in different contexts. Word association methods demonstrate cognitively-grounded differences in the specific use of language, and reveal difficult-to-articulate differences in syntax that reflect topical mental maps (Cardak, 2009; Gravino *et al.*, 2012). The results of a word association analysis can be represented and analyzed as a network, with key terms [either pre-selected Glossary terms (blue points), or author-choice terms (red points)] as nodes and correlated use of these terms (i.e., words most frequently placed in close prox-imity of the key terms above the 75th percentile) represented as edges. Therefore, this approach estimates how authors studying different ecological contexts convey language related to stochasticity. If differences are found between the word association networks produced in different contexts, this provides evidence that ecologists are not using the term stochasticity in a synthetic manner, which hampers our ability to make significant, unified progress in the field of stochastic ecology.

Methods

To determine the context surrounding different uses of "stochasticity" within population and community ecology, we searched the recent literature for journal articles related to stochasticity. We limited our word association analysis to focus on the comparison between the three forms of stochasticity encountered in ecological systems (Fig. 1) - demographic stochasticity (using search terms: "demographic stochastic*" AND "ecology"),

environmental stochasticity (using search terms: “environmental stochastic*” AND “ecology”), and measurement error (using search terms: “measurement error” OR “observation* error” AND “ecology”). We applied our search terms to the TOPIC and TITLE fields in Web of Science and limited journal articles to the recent literature from 2010-2015. This search yielded 46, 43, and 55 relevant articles respectively (Table S1). We removed less than 5 papers per type of stochasticity, and only if the results were not regarding community ecology specifically, or clearly did not pertain to ecology.

Word association analysis was performed in R 3.2.4 using the tm package (Feinerer & Hornik, 2017; Meyer *et al.*, 2008). Common English stop words, as delineated in the tm package, and other words that did not convey any meaning to our analysis were removed (e.g. effects, important, given, figure). We grouped articles by form of stochasticity (demographic, environmental, measurement/observational error), and calculated correlations between all words in each article but only considered pairs where at least one word was related to stochasticity (Table S2 – a slightly extended version of Box 1). The strength of correlations between word pairs was defined by distance – words closer in the text had stronger correlations than words farther from each other. To construct the networks, we made a list of edges (i.e. all associations between word pairs) and created distributions of correlation coefficients for each pair within the list across all papers within a group. To reduce the complexity of each network, we removed words that had abundances across all papers that fell below the 95th percentile. Further, relationships between words were removed if the correlation coefficient fell below the 75th percentile. Networks were constructed and plotted using the igraph and GGally packages (Csardi & Nepusz, 2006; Schloerke *et al.*, 2016).

Results

Assuming “stochasticity” has one definition, then finding differences in the associations between words across contexts provides evidence that the field does not use the term synthetically (Cattuto *et al.*, 2009; Gravino *et al.*, 2012). We find language concerning demographic stochasticity (Fig. S1) tends to be more inclusive (i.e. a larger, more connected network of terms), and includes the key concepts of uncertainty, random, and variation. In comparison, the environmental stochasticity (Fig. S2) word association network has a smaller main component (i.e. less words connected in a single module, or cluster), but contains the key concepts of probability and noise. From our networks, we observe that both demographic and environmental stochasticity are invoked at both population and community levels, exemplifying that ecologists are interested in how stochastic processes scale to alter both levels of ecological organization. Surprisingly, none of the contexts had “distribution” – which is central to the concept of stochasticity – within the module containing “stochasticity” or “error”, though the term did appear in both the demographic stochasticity and measurement error (Fig. S3) networks.

While the outcomes of this word association analysis can vary based on the papers chosen and the inclusion parameters selected, the purpose of this work is to demonstrate that

there is inconsistency across subfields of ecology when it comes to thinking about stochasticity. Our work illustrates the interest in stochastic processes across ecology, while also emphasizing the need for cohesive vocabulary within the field that ecologists can use to communicate across disciplines and scales.

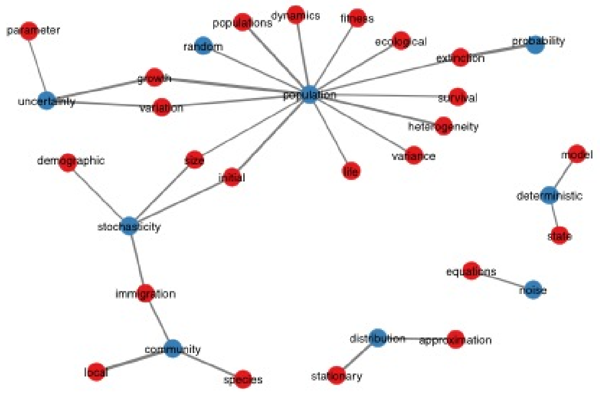

Figure S1: Demographic Stochasticity

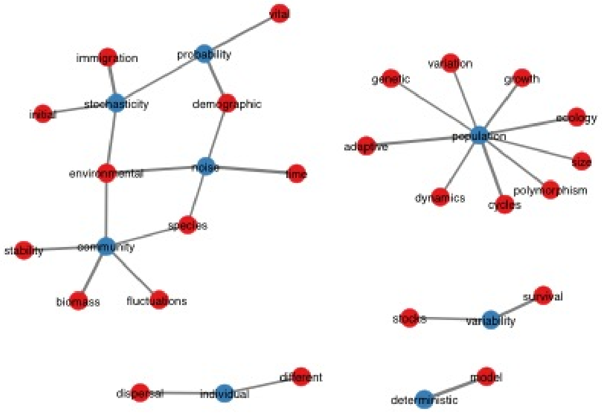

Figure S2: Environmental Stochasticity

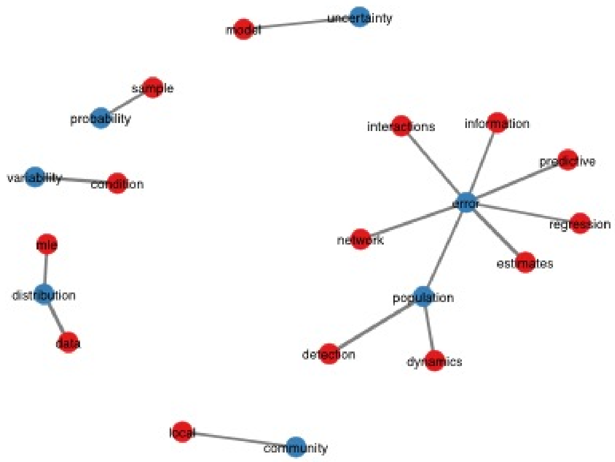

Figure S3: Measurement/Observation Error

Table S1. Papers used in word association analysis

type	citations
Demographic	Bansaye & Lambert 2013; Bessa-Gomes *et al.* 2010; Billiard & Tran 2012; Bolnick *et al.* 2011; Bonachela *et al.* 2012; Carrara *et al.* 2015; Carroll & Nisbet 2015; Carval *et al.* 2015; Cassey *et al.* 2014; Chisholm *et al.* 2014; Dochtermann & Gienger 2012; zu Dohna & Pineda-Krch 2010; Drake *et al.* 2011; Dugaw & Ram 2011; Erickson *et al.* 2015; Espinosa *et al.* 2012; Evans *et al.* 2010; Gascuel *et al.* 2015; Gibson & Wilson 2013; Giometto *et al.* 2014; Golestani *et al.* 2012; Griffen *et al.* 2012; Gustafsson & Sternad 2013; Haegeman & Loreau 2011; Hammond & Niklas 2011; Haney *et al.* 2015; He *et al.* 2012; Jacobs & Sluckin 2015; Jongejans *et al.* 2011; Kendall *et al.* 2011; Kolpas & Nisbet 2010; Kramer & Drake 2014; Law *et al.* 2015; Lin *et al.* 2015; Linnerud *et al.* 2013; Martín *et al.* 2015; M'Gonigle & Greenspoon 2014; Münkemüller *et al.* 2011; Mutshinda & O'Hara 2011; Pedruski *et al.* 2015; Poisot *et al.* 2014; Sæther *et al.* 2013; Shipley 2014; Shipley *et al.* 2012; Tanentzap *et al.* 2013; Yamamichi *et al.* 2014
Environmental	Armstrong & Ewen 2013; Aubry *et al.* 2010; Bjørkvoll *et al.* 2012; Bode *et al.* 2011; Bowler & Benton 2011; Campioni *et al.* 2010; Cassey *et al.* 2014; Cosentino *et al.* 2010; Crawford *et al.* 2015; Cronin & Reeve 2014; Cumming *et al.* 2012; Delgado *et al.* 2010; Dugaw & Ram 2011; Epperson *et al.* 2010; Evans *et al.* 2010; Ferguson & Ponciano 2014; Fowler & Ruokolainen 2013; Giometto *et al.* 2015; Gustafsson & Sternad 2013; Harrison *et al.* 2011; Hostetler *et al.* 2012; Kalyuzhny *et al.* 2014; Kessler *et al.* 2015; Kiernan & Moyle 2012; Lahoz-Monfort *et al.* 2011; Law *et al.* 2015; Linnerud *et al.* 2013; Menu *et al.* 2010; Ohlberger *et al.* 2014; Oppel *et al.* 2014; Paiva *et al.* 2013a,b; Péron & Koons 2013; Robert *et al.* 2014; Sæther *et al.* 2013; Scheihing *et al.* 2011; Shefferson *et al.* 2012; Sinclair *et al.* 2013; Soutullo *et al.* 2013; Suhonen *et al.* 2010; Svardal *et al.* 2015; Tilles & Petrovskii 2015; Wilson White *et al.* 2014
Measurement	Al-Wathiqui & Rodríguez 2011; Bird *et al.* 2014; Bjørkvoll *et al.* 2012; Borchers *et al.* 2015; Buoro *et al.* 2012; Burgman *et al.* 2012; Cardoso & Gomes 2015; Chakraborty *et al.* 2010; Connette *et al.* 2015; Damgaard 2014; Dechaume-Moncharmont *et al.* 2011; Denham *et al.* 2011; Dingemanse & Dochtermann 2013; Dugaw & Ram 2011; Dujon *et al.* 2014; Duncan & Vesk 2013; Eaton & Link 2011; Farmer *et al.* 2014; Fieberg & Ditmer 2012; Fletcher & Dixon 2012; Foster *et al.* 2012; Freckleton 2011; Garamszegi & Møller 2012; Goodenough *et al.* 2010; Gould *et al.* 2014; Hefley *et al.* 2014, 2017; Hoenner *et al.* 2012; Jabot 2015; Karban & De Valpine 2010; Kibler *et al.* 2011; Lambert *et al.* 2012; Laube & Purves 2011; Martin *et al.* 2013; McClintock *et al.* 2010, 2013; McGeoch *et al.* 2012; Merow *et al.* 2011; Millar *et al.* 2014; Miller *et al.* 2012; Mutshinda & O'Hara 2011; Novak *et al.* 2011; Ohlberger *et al.* 2014; Queenborough *et al.* 2011; Recio *et al.* 2013; Ross *et al.* 2012; Ross 2012; Sigourney *et al.* 2012; Simard *et al.* 2012; Stoklosa *et al.* 2015; Suprenand *et al.* 2015; Taubert *et al.* 2013; Van Moorter *et al.* 2010; Warton 2015; Winterhalder *et al.* 2010

Table S2. Terms used in word association analysis

Terms
stochastic*
random
deterministic
distribution
variabil*
uncertainty
predict*
error
noise
autocorrelation
individual
population
community

References

Al-Wathiqui, N. & Rodríguez, R.L. (2011). Allometric slopes not underestimated by ordinary least squares regression: a case study with enchenopa treehoppers (hemiptera: Membracidae). *Annals of the Entomological Society of America*, 104, 562–566.

Armstrong, D.P. & Ewen, J.G. (2013). Consistency, continuity and creativity: long-term studies of population dynamics on tiritiri matangi island. *New Zealand Journal of Ecology*, 37, 288–297.

Aubry, A., Bécart, E., Davenport, J. & Emmerson, M.C. (2010). Estimation of survival rate and extinction probability for stage-structured populations with overlapping life stages. *Population Ecology*, 52, 437–450.

Bansaye, V. & Lambert, A. (2013). New approaches to source–sink metapopulations decoupling demography and dispersal. *Theoretical Population Biology*, 88, 31–46.

Bessa-Gomes, C., Legendre, S. & Clobert, J. (2010). Discrete two-sex models of population dynamics: on modelling the mating function. *Acta Oecologica*, 36, 439–445.

Billiard, S. & Tran, V.C. (2012). A general stochastic model for sporophytic self-incompatibility. *Journal of Mathematical Biology*, 64, 163–210.

Bird, T.J., Bates, A.E., Lefcheck, J.S., Hill, N.A., Thomson, R.J., Edgar, G.J., Stuart-Smith, R.D., Wotherspoon, S., Krkosek, M., Stuart-Smith, J.F. *et al.* (2014). Statistical solutions for error and bias in global citizen science datasets. *Biological Conservation*, 173, 144–154.

Bjørkvoll, E., Grøtan, V., Aanes, S., Sæther, B.E., Engen, S. & Aanes, R. (2012). Stochastic population dynamics and life-history variation in marine fish species. *The American Naturalist*, 180, 372–387.

Bode, M., Bode, L. & Armsworth, P.R. (2011). Different dispersal abilities allow reef fish to coexist. *Proceedings of the National Academy of Sciences*, 108, 16317–16321.

Bolnick, D.I., Amarasekare, P., Araújo, M.S., Bürger, R., Levine, J.M., Novak, M., Rudolf, V.H., Schreiber, S.J., Urban, M.C. & Vasseur, D.A. (2011). Why intraspecific trait variation matters in community ecology. *Trends in Ecology & Evolution*, 26, 183–192.

Bonachela, J.A., Muñoz, M.A. & Levin, S.A. (2012). Patchiness and demographic noise in three ecological examples. *Journal of Statistical Physics*, 148, 724–740.

Borchers, D., Stevenson, B., Kidney, D., Thomas, L. & Marques, T. (2015). A unifying model for capture–recapture and distance sampling surveys of wildlife populations. *Journal of the American Statistical Association*, 110, 195–204.

Bowler, D.E. & Benton, T.G. (2011). Testing the interaction between environmental variation and dispersal strategy on population dynamics using a soil mite experimental system. *Oecologia*, 166, 111–119.

Buoro, M., Prévost, E. & Gimenez, O. (2012). Digging through model complexity: using hierarchical models to uncover evolutionary processes in the wild. *Journal of Evolutionary Biology*, 25, 2077–2090.

Burgman, M., Franklin, J., Hayes, K.R., Hosack, G.R., Peters, G.W. & Sisson, S.A. (2012). Modeling extreme risks in ecology. *Risk Analysis*, 32, 1956–1966.

Campioni, L., Delgado, M.D. & Penteriani, V. (2010). Social status influences microhabitat selection: breeder and floater eagle owls bubo bubo use different post sites. *Ibis*, 152, 569–579.

Cardak, O. (2009). The determiniation of the knowledge level of science students on energy flow through a word association test. *Energy Education Science and Technology Part B-Social and Educational Studies*, 1, 139–155.

Cardoso, G.C. & Gomes, A.C.R. (2015). Using reflectance ratios to study animal coloration. *Evolutionary Biology*, 42, 387–394.

Carrara, F., Giometto, A., Seymour, M., Rinaldo, A. & Altermatt, F. (2015). Inferring species interactions in ecological communities: a comparison of methods at different levels of complexity. *Methods in Ecology and Evolution*, 6, 895–906.

Carroll, I.T. & Nisbet, R.M. (2015). Departures from neutrality induced by niche and relative fitness differences. *Theoretical Ecology*, 8, 449–465.

Carval, D., Perrin, B., Duyck, P.F. & Tixier, P. (2015). Local and neighboring patch conditions alter sex-specific movement in banana weevils. *Ecology and Evolution*, 5, 5735–5743.

Cassey, P., Prowse, T.A. & Blackburn, T.M. (2014). A population model for predicting the successful establishment of introduced bird species. *Oecologia*, 175, 417–428.

Cattuto, C., Barrat, A., Baldassarri, A., Schehr, G. & Loreto, V. (2009). Collective dynamics of social annotation. *Proceedings of the National Academy of Sciences*, 106, 10511–10515.

Chakraborty, A., Gelfand, A.E., Wilson, A.M., Latimer, A.M. & Silander Jr, J.A. (2010). Modeling large scale species abundance with latent spatial processes. *The Annals of Applied Statistics*, pp. 1403–1429.

Chisholm, R.A., Condit, R., Rahman, K.A., Baker, P.J., Bunyavejchewin, S., Chen, Y.Y., Chuyong, G., Dattaraja, H., Davies, S., Ewango, C.E. *et al.* (2014). Temporal variability of forest communities: empirical estimates of population change in 4000 tree species. *Ecology Letters*, 17, 855–865.

Connette, G.M., Crawford, J.A. & Peterman, W.E. (2015). Climate change and shrinking salamanders: alternative mechanisms for changes in plethodontid salamander body size. *Global Change Biology*, 21, 2834–2843.

Cosentino, B.J., Schooley, R.L. & Phillips, C.A. (2010). Wetland hydrology, area, and isolation influence occupancy and spatial turnover of the painted turtle, chrysemys picta. *Landscape Ecology*, 25, 1589–1600.

Crawford, M., Davies, S. & Griffith, A. (2015). Predicting metapopulation responses of a tidal wetland annual to environmental stochasticity and water dispersal through an individual-based model. *Ecological Modelling*, 316, 217–229.

Cronin, J.T. & Reeve, J.D. (2014). An integrative approach to understanding host–parasitoid population dynamics in real landscapes. *Basic and Applied Ecology*, 15, 101–113.

Csardi, G. & Nepusz, T. (2006). The igraph software package for complex network research. *InterJournal, Complex Systems*, 1695, 1–9.

Cumming, G.S., Gaidet, N. & Ndlovu, M. (2012). Towards a unification of movement ecology and biogeography: conceptual framework and a case study on afrotropical ducks. *Journal of Biogeography*, 39, 1401–1411.

Damgaard, C. (2014). Estimating mean plant cover from different types of cover data: a coherent statistical framework. *Ecosphere*, 5, 1–7.

Dechaume-Moncharmont, F.X., Monceau, K. & Cezilly, F. (2011). Sexing birds using discriminant function analysis: a critical appraisal. *The Auk*, 128, 78–86.

Delgado, M.d.M., Penteriani, V., Revilla, E. & Nams, V.O. (2010). The effect of phenotypic traits and external cues on natal dispersal movements. *Journal of Animal Ecology*, 79, 620–632.

Denham, R.J., Falk, M.G. & Mengersen, K.L. (2011). The bayesian conditional independence model for measurement error: applications in ecology. *Environmental and Ecological Statistics*, 18, 239–255.

Dingemanse, N.J. & Dochtermann, N.A. (2013). Quantifying individual variation in behaviour: mixed-effect modelling approaches. *Journal of Animal Ecology*, 82, 39–54.

Dochtermann, N.A. & Gienger, C.M. (2012). Individual variability in life-history traits drives population size stability. *Current Zoology*, 58, 358–362.

zu Dohna, H. & Pineda-Krch, M. (2010). Fitting parameters of stochastic birth–death models to metapopulation data. *Theoretical Population Biology*, 78, 71–76.

Drake, J.M., Shapiro, J. & Griffen, B.D. (2011). Experimental demonstration of a two-phase population extinction hazard. *Journal of the Royal Society Interface*, 8, 1472–1479.

Dugaw, C.J. & Ram, K. (2011). Individual heterogeneity in mortality mediates long-term persistence of a seasonal microparasite. *Oecologia*, 166, 317–325.

Dujon, A.M., Lindstrom, R.T. & Hays, G.C. (2014). The accuracy of fastloc-gps locations and implications for animal tracking. *Methods in Ecology and Evolution*, 5, 1162–1169.

Duncan, D.H. & Vesk, P.A. (2013). Examining change over time in habitat attributes using bayesian reinterpretation of categorical assessments. *Ecological Applications*, 23, 1277–1287.

Eaton, M.J. & Link, W.A. (2011). Estimating age from recapture data: integrating incremental growth measures with ancillary data to infer age-at-length. *Ecological Applications*, 21, 2487–2497.

Epperson, B.K., McRae, B.H., Scribner, K., Cushman, S.A., Rosenberg, M.S., Fortin, M.J., James, P., Murphy, M., Manel, S., Legendre, P. *et al.* (2010). Utility of computer simulations in landscape genetics. *Molecular Ecology*, 19, 3549–3564.

Erickson, R.A., Eager, E.A., Stanton, J.C., Beston, J.A., Diffendorfer, J.E. & Thogmartin, W.E. (2015). Assessing local population vulnerability with branching process models: an application to wind energy development. *Ecosphere*, 6, 1–14.

Espinosa, M.I., Bertin, A., Gómez, J., Mejia, F., Guerra, M., Baez, L., Gouin, N. & Patiño, E. (2012). A three-year mark-recapture study in a remnant population of crocodylus acutus cuvier in portete bay (guajira, colombia). *Gayana*, 76, 52.

Evans, M.E., Holsinger, K.E. & Menges, E.S. (2010). Fire, vital rates, and population viability: a hierarchical bayesian analysis of the endangered florida scrub mint. *Ecological Monographs*, 80, 627–649.

Farmer, R.G., Leonard, M.L., Mills Flemming, J.E. & Anderson, S.C. (2014). Observer aging and long-term avian survey data quality. *Ecology and Evolution*, 4, 2563–2576.

Feinerer, I. & Hornik, K. (2017). tm: Text mining package. r package version 0.7-1.

Ferguson, J.M. & Ponciano, J.M. (2014). Predicting the process of extinction in experimental microcosms and accounting for interspecific interactions in single-species time series. *Ecology Letters*, 17, 251–259.

Fieberg, J. & Ditmer, M. (2012). Understanding the causes and consequences of animal movement: a cautionary note on fitting and interpreting regression models with time-dependent covariates. *Methods in Ecology and Evolution*, 3, 983–991.

Fletcher, D. & Dixon, P.M. (2012). Modelling data from different sites, times or studies: weighted vs. unweighted regression. *Methods in Ecology and Evolution*, 3, 168–176.

Foster, S.D., Shimadzu, H. & Darnell, R. (2012). Uncertainty in spatially predicted covariates: is it ignorable? *Journal of the Royal Statistical Society: Series C (Applied Statistics)*, 61, 637–652.

Fowler, M.S. & Ruokolainen, L. (2013). Colonization, covariance and colour: Environmental and ecological drivers of diversity–stability relationships. *Journal of Theoretical Biology*, 324, 32–41.

Freckleton, R.P. (2011). Dealing with collinearity in behavioural and ecological data: model averaging and the problems of measurement error. *Behavioral Ecology and Sociobiology*, 65, 91–101.

Garamszegi, L.Z. & Møller, A.P. (2012). Untested assumptions about within-species sample size and missing data in interspecific studies. *Behavioral Ecology and Sociobiology*, 66, 1363–1373.

Gascuel, F., Ferrière, R., Aguilée, R. & Lambert, A. (2015). How ecology and landscape dynamics shape phylogenetic trees. *Systematic Biology*, 64, 590–607.

Gibson, W.T. & Wilson, W.G. (2013). Individual-based chaos: Extensions of the discrete logistic model. *Journal of Theoretical Biology*, 339, 84–92.

Giometto, A., Formentin, M., Rinaldo, A., Cohen, J.E. & Maritan, A. (2015). Sample and population exponents of generalized taylor?s law. *Proceedings of the National Academy of Sciences*, 112, 7755–7760.

Giometto, A., Rinaldo, A., Carrara, F. & Altermatt, F. (2014). Emerging predictable features of replicated biological invasion fronts. *Proceedings of the National Academy of Sciences*, 111, 297–301.

Golestani, A., Gras, R. & Cristescu, M. (2012). Speciation with gene flow in a heterogeneous virtual world: can physical obstacles accelerate speciation? *Proceedings of the Royal Society of London B: Biological Sciences*, 279, 3055–3064.

Goodenough, A.E., Stafford, R., Catlin-Groves, C.L., Smith, A.L. & Hart, A.G. (2010). Within-and among-observer variation in measurements of animal biometrics and their influence on accurate quantification of common biometric-based condition indices. *Annales Zoologici Fennici*, 47, 323–334.

Gould, S.F., Beeton, N.J., Harris, R., Hutchinson, M.F., Lechner, A.M., Porfirio, L.L. & Mackey, B.G. (2014). A tool for simulating and communicating uncertainty when modelling species distributions under future climates. *Ecology and Evolution*, 4, 4798–4811.

Gravino, P., Servedio, V.D., Barrat, A. & Loreto, V. (2012). Complex structures and semantics in free word association. *Advances in Complex Systems*, 15, 1250054.

Griffen, B.D., Altman, I., Bess, B.M., Hurley, J. & Penfield, A. (2012). The role of foraging in the success of invasive asian shore crabs in new england. *Biological Invasions*, 14, 2545–2558.

Gustafsson, L. & Sternad, M. (2013). When can a deterministic model of a population system reveal what will happen on average? *Mathematical Biosciences*, 243, 28–45.

Haegeman, B. & Loreau, M. (2011). A mathematical synthesis of niche and neutral theories in community ecology. *Journal of Theoretical Biology*, 269, 150–165.

Hammond, S.T. & Niklas, K.J. (2011). Computer simulations of plant biodiversity in stable and unstable environments: A test of the neutral biodiversity theory. *Journal of Biological Systems*, 19, 1–17.

Haney, S., Cattivera, M. & Siepielski, A.M. (2015). Rare niches and the ecological equivalence of species. *Theoretical Ecology*, 8, 491–503.

Harrison, P.J., Hanski, I. & Ovaskainen, O. (2011). Bayesian state-space modeling of metapopulation dynamics in the glanville fritillary butterfly. *Ecological Monographs*, 81, 581–598.

He, F., Zhang, D.Y. & Lin, K. (2012). Coexistence of nearly neutral species. *Journal of Plant Ecology*, 5, 72–81.

Hefley, T.J., Baasch, D.M., Tyre, A.J. & Blankenship, E.E. (2014). Correction of location errors for presence-only species distribution models. *Methods in Ecology and Evolution*, 5, 207–214.

Hefley, T.J., Tyre, A.J. & Blankenship, E.E. (2017). Reprint of: Fitting population growth models in the presence of measurement and detection error. *Ecological Modelling*, 359, 461–467.

Hoenner, X., Whiting, S.D., Hindell, M.A. & McMahon, C.R. (2012). Enhancing the use of argos satellite data for home range and long distance migration studies of marine animals. *PLoS One*, 7, e40713.

Hostetler, J.A., Kneip, E., Van Vuren, D.H. & Oli, M.K. (2012). Stochastic population dynamics of a montane ground-dwelling squirrel. *PloS One*, 7, e34379.

Jabot, F. (2015). Why preferring parametric forecasting to nonparametric methods? *Journal of Theoretical Biology*, 372, 205–210.

Jacobs, G.S. & Sluckin, T.J. (2015). Long-range dispersal, stochasticity and the broken accelerating wave of advance. *Theoretical Population Biology*, 100, 39–55.

Jongejans, E., Allen, M.R., Leib, A.E., Marchetto, K.M., Pedatella, N.M., Peterson-Smith, J., Rauschert, E.S.J., Ruggiero, D.C., Russo, L.A., Ruth, L.E., Sezen, Z., Skarpaas, O., Teller, B.J., Warg, L.A., Yang, W.S., Zhang, R. & Shea, K. (2011). Spatial dynamics of invasive carduus thistles. *19th International Congress on Modelling and Simulation*.

Kalyuzhny, M., Schreiber, Y., Chocron, R., Flather, C.H., Kadmon, R., Kessler, D.A. & Shnerb, N.M. (2014). Temporal fluctuation scaling in populations and communities. *Ecology*, 95, 1701–1709.

Karban, R. & De Valpine, P. (2010). Population dynamics of an arctiid caterpillar–tachinid parasitoid system using state-space models. *Journal of Animal Ecology*, 79, 650–661.

Kendall, B.E., Fox, G.A., Fujiwara, M. & Nogeire, T.M. (2011). Demographic heterogeneity, cohort selection, and population growth. *Ecology*, 92, 1985–1993.

Kessler, D., Suweis, S., Formentin, M. & Shnerb, N.M. (2015). Neutral dynamics with environmental noise: Age-size statistics and species lifetimes. *Physical Review E*, 92, 022722.

Kibler, K., Tullos, D. & Kondolf, G. (2011). Learning from dam removal monitoring: challenges to selecting experimental design and establishing significance of outcomes. *River Research and Applications*, 27, 967–975.

Kiernan, J.D. & Moyle, P.B. (2012). Flows, droughts, and aliens: factors affecting the fish assemblage in a sierra nevada, california, stream. *Ecological Applications*, 22, 1146–1161.

Kolpas, A. & Nisbet, R.M. (2010). Effects of demographic stochasticity on population persistence in advective media. *Bulletin of Mathematical Biology*, 72, 1254–1270.

Kramer, A.M. & Drake, J.M. (2014). Time to competitive exclusion. *Ecosphere*, 5, 1–16.

Lahoz-Monfort, J.J., Morgan, B.J., Harris, M.P., Wanless, S. & Freeman, S.N. (2011). A capture–recapture model for exploring multi-species synchrony in survival. *Methods in Ecology and Evolution*, 2, 116–124.

Lambert, M.R., Yasuda, C.M. & Todd, B.D. (2012). Evaluation of a photographic technique for estimating body size in lizards from a distance. *Herpetological Conservation and Biology*, 7, 83–88.

Laube, P. & Purves, R.S. (2011). How fast is a cow? cross-scale analysis of movement data. *Transactions in GIS*, 15, 401–418.

Law, P.R., Fike, B. & Lent, P.C. (2015). Dynamics of an expanding black rhinoceros (*Diceros bicornis minor*) population. *European Journal of Wildlife Research*, 61, 601–609.

Lin, Y.T., Kim, H. & Doering, C.R. (2015). Demographic stochasticity and evolution of dispersion i. spatially homogeneous environments. *Journal of Mathematical Biology*, 70, 647–678.

Linnerud, M., Sæther, B.E., Grøtan, V., Engen, S., Noble, D.G. & Freckleton, R.P. (2013). Interspecific differences in stochastic population dynamics explains variation in taylor's temporal power law. *Oikos*, 122, 1207–1216.

Martin, J., Festa-Bianchet, M., Côté, S. & Blumstein, D. (2013). Detecting between-individual differences in hind-foot length in populations of wild mammals. *Canadian Journal of Zoology*, 91, 118–123.

Martín, P.V., Bonachela, J.A., Levin, S.A. & Muñoz, M.A. (2015). Eluding catastrophic shifts. *Proceedings of the National Academy of Sciences*, 112, E1828–E1836.

McClintock, B.T., Nichols, J.D., Bailey, L.L., MacKenzie, D.I., Kendall, W., Franklin, A.B. *et al.* (2010). Seeking a second opinion: uncertainty in disease ecology. *Ecology Letters*, 13, 659–674.

McClintock, B.T., Russell, D.J., Matthiopoulos, J. & King, R. (2013). Combining individual animal movement and ancillary biotelemetry data to investigate population-level activity budgets. *Ecology*, 94, 838–849.

McGeoch, M.A., Spear, D., Kleynhans, E.J. & Marais, E. (2012). Uncertainty in invasive alien species listing. *Ecological Applications*, 22, 959–971.

Menu, F., Ginoux, M., Rajon, E., Lazzari, C.R. & Rabinovich, J.E. (2010). Adaptive developmental delay in chagas disease vectors: an evolutionary ecology approach. *PLoS Neglected Tropical Diseases*, 4, e691.

Merow, C., Latimer, A.M. & Silander, J.A. (2011). Can entropy maximization use functional traits to explain species abundances? a comprehensive evaluation. *Ecology*, 92, 1523–1537.

Meyer, D., Hornik, K. & Feinerer, I. (2008). Text mining infrastructure in r. *Journal of Statistical Software*, 25, 1–54.

M'Gonigle, L.K. & Greenspoon, P.B. (2014). Allee effects and species co-existence in an environment where resource abundance varies. *Journal of Theoretical Biology*, 361, 61–68.

Millar, C.P., Jardim, E., Scott, F., Osio, G.C., Mosqueira, I. & Alzorriz, N. (2014). Model averaging to streamline the stock assessment process. *ICES Journal of Marine Science*, 72, 93–98.

Miller, D.A., Talley, B.L., Lips, K.R. & Campbell Grant, E.H. (2012). Estimating patterns and drivers of infection prevalence and intensity when detection is imperfect and sampling error occurs. *Methods in Ecology and Evolution*, 3, 850–859.

Münkemüller, T., Travis, M., Burton, O., Schiffers, K. & Johst, K. (2011). Density-regulated population dynamics and conditional dispersal alter the fate of mutations occurring at the front of an expanding population. *Heredity*, 106, 678.

Mutshinda, C.M. & O'Hara, R.B. (2011). Integrating the niche and neutral perspectives on community structure and dynamics. *Oecologia*, 166, 241–251.

Novak, M., Wootton, J.T., Doak, D.F., Emmerson, M., Estes, J.A. & Tinker, M.T. (2011). Predicting community responses to perturbations in the face of imperfect knowledge and network complexity. *Ecology*, 92, 836–846.

Ohlberger, J., Rogers, L.A. & Stenseth, N.C. (2014). Stochasticity and determinism: how density-independent and density-dependent processes affect population variability. *PloS One*, 9, e98940.

Oppel, S., Hilton, G., Ratcliffe, N., Fenton, C., Daley, J., Gray, G., Vickery, J. & Gibbons, D. (2014). Assessing population viability while accounting for demographic and environmental uncertainty. *Ecology*, 95, 1809–1818.

Paiva, V.H., Geraldes, P., Marques, V., Rodriguez, R., Garthe, S. & Ramos, J.A. (2013a). Effects of environmental variability on different trophic levels of the north atlantic food web. *Marine Ecology Progress Series*, 477, 15–28.

Paiva, V.H., Geraldes, P., Ramirez, I., Werner, A.C., Garthe, S. & Ramos, J.A. (2013b). Overcoming difficult times: the behavioural resilience of a marine predator when facing environmental stochasticity. *Marine Ecology Progress Series*, 486, 277–288.

Pedruski, M.T., Fussmann, G.F. & Gonzalez, A. (2015). Predicting the outcome of competition when fitness inequality is variable. *Royal Society Open Science*, 2, 150274.

Péron, G. & Koons, D.N. (2013). Intra-guild interactions and projected impact of climate and land use changes on north american pochard ducks. *Oecologia*, 172, 1159–1165.

Poisot, T., Bever, J.D., Thrall, P.H. & Hochberg, M.E. (2014). Dispersal and spatial heterogeneity allow coexistence between enemies and protective mutualists. *Ecology and Evolution*, 4, 3841–3850.

Queenborough, S.A., Burnet, K.M., Sutherland, W.J., Watkinson, A.R. & Freckleton, R.P. (2011). From meso-to macroscale population dynamics: a new density-structured approach. *Methods in Ecology and Evolution*, 2, 289–302.

Recio, M.R., Mathieu, R., Latham, M.C., Latham, A.D.M. & Seddon, P.J. (2013). Quantifying fine-scale resource selection by introduced european hedgehogs (erinaceus europaeus) in ecologically sensitive areas. *Biological invasions*, 15, 1807–1818.

Robert, A., Paiva, V.H., Bolton, M., Jiguet, F. & Bried, J. (2014). Nest fidelity is driven by multi-scale information in a long-lived seabird. *Proceedings of the Royal Society of London B: Biological Sciences*, 281, 20141692.

Ross, B.E., Hooten, M.B. & Koons, D.N. (2012). An accessible method for implementing hierarchical models with spatio-temporal abundance data. *PLoS One*, 7, e49395.

Ross, J. (2012). On parameter estimation in population models iii: Time-inhomogeneous processes and observation error. *Theoretical Population Biology*, 82, 1–17.

Sæther, B.E., Coulson, T., Grøtan, V., Engen, S., Altwegg, R., Armitage, K.B., Barbraud, C., Becker, P.H., Blumstein, D.T., Dobson, F.S., Festa-Bianchet, M., Gailard, J.M., Jenkins, A., Jones, C., Nicoll, M.A.C., Norris, K., Oli, M.K., Ozgul, A. & Weimerskirch, H. (2013). How life history influences population dynamics in fluctuating environments. *The American Naturalist*, 182, 743–759.

Scheihing, R., Cardenas, L., Labarca, P. & Nespolo, R.F. (2011). Spatial and temporal analysis of the genetic diversity and population structure of a freshwater ostracod from the high andean plateau. *Evolutionary Ecology Research*, 13, 91–104.

Schloerke, B., Crowley, J., Cook, D., Briatte, F., Marbach, M., E., T., Elberg, A. & Larmarange, J. (2016). Ggally: Extension to 'ggplot2'. r package version 1.2.0. Tech. rep.

Shefferson, R.P., Kull, T., Tali, K. & Kellett, K.M. (2012). Linking vegetative dormancy to fitness in two long-lived herbaceous perennials. *Ecosphere*, 3, 1–19.

Shipley, B. (2014). Measuring and interpreting trait-based selection versus meta-community effects during local community assembly. *Journal of Vegetation Science*, 25, 55–65.

Shipley, B., Paine, C. & Baraloto, C. (2012). Quantifying the importance of local niche-based and stochastic processes to tropical tree community assembly. *Ecology*, 93, 760–769.

Sigourney, D.B., Munch, S.B. & Letcher, B.H. (2012). Combining a bayesian nonparametric method with a hierarchical framework to estimate individual and temporal variation in growth. *Ecological Modelling*, 247, 125–134.

Simard, M.A., Côté, S.D., Gingras, A. & Coulson, T. (2012). Tests of density dependence using indices of relative abundance in a deer population. *Oikos*, 121, 1351–1363.

Sinclair, A., Metzger, K.L., Fryxell, J.M., Packer, C., Byrom, A.E., Craft, M.E., Hampson, K., Lembo, T., Durant, S.M., Forrester, G.J. *et al.* (2013). Asynchronous food-web pathways could buffer the response of serengeti predators to el niño southern oscillation. *Ecology*, 94, 1123–1130.

Soutullo, A., López-López, P., Cortés, G.D., Urios, V. & Ferrer, M. (2013). Exploring juvenile golden eagles' dispersal movements at two different temporal scales. *Ethology Ecology & Evolution*, 25, 117–128.

Stoklosa, J., Daly, C., Foster, S.D., Ashcroft, M.B. & Warton, D.I. (2015). A climate of uncertainty: accounting for error in climate variables for species distribution models. *Methods in Ecology and Evolution*, 6, 412–423.

Suhonen, J., Hilli-Lukkarinen, M., Korkeamaeki, E., Kuitunen, M., Kullas, J., Penttinen, J. & Salmela, J. (2010). Local extinction of dragonfly and damselfly populations in low- and high-quality habitat patches. *Conservation Biology*, 24, 1148–1153.

Suprenand, P.M., Drexler, M., Jones, D.L. & Ainsworth, C.H. (2015). Strategic assessment of fisheries independent monitoring programs in the gulf of mexico. *PloS One*, 10, e0120929.

Svardal, H., Rueffler, C. & Hermisson, J. (2015). A general condition for adaptive genetic polymorphism in temporally and spatially heterogeneous environments. *Theoretical Population Biology*, 99, 76–97.

Tanentzap, A.J., Lee, W.G. & Schulz, K.A.C. (2013). Niches drive peaked and positive relationships between diversity and disturbance in natural ecosystems. *Ecosphere*, 4, 1–28.

Taubert, F., Hartig, F., Dobner, H.J. & Huth, A. (2013). On the challenge of fitting tree size distributions in ecology. *PloS one*, 8, e58036.

Tilles, P.F. & Petrovskii, S.V. (2015). Statistical mechanics of animal movement: Animals's decision-making can result in superdiffusive spread. *Ecological Complexity*, 22, 86–92.

Van Moorter, B., Visscher, D.R., Jerde, C.L., Frair, J.L. & Merrill, E.H. (2010). Identifying movement states from location data using cluster analysis. *Journal of Wildlife Management*, 74, 588–594.

Vellend, M., Srivastava, D.S., Anderson, K.M., Brown, C.D., Jankowski, J.E., Kleynhans, E.J., Kraft, N.J.B., Letaw, A.D., Macdonald, A.A.M., Maclean, J.E., Myers-Smith, I.H., Norris, A.R. & Xue, X. (2014). Assessing the relative importance of neutral stochasticity in ecological communities. *Oikos*, 123, 1420–1430.

Warton, D.I. (2015). New opportunities at the interface between ecology and statistics. *Methods in Ecology and Evolution*, 6, 363–365.

Wilson White, J., Botsford, L.W., Hastings, A. & Holland, M.D. (2014). Stochastic models reveal conditions for cyclic dominance in sockeye salmon populations. *Ecological Monographs*, 84, 69–90.

Winterhalder, B., Kennett, D.J., Grote, M.N. & Bartruff, J. (2010). Ideal free settlement of california's northern channel islands. *Journal of Anthropological Archaeology*, 29, 469–490.

Yamamichi, M., Yoshida, T. & Sasaki, A. (2014). Timing and propagule size of invasion determine its success by a time-varying threshold of demographic regime shift. *Ecology*, 95, 2303–2315.
