# Supplementary material for: Integrating the underlying structure of stochasticity into community ecology
Source: Ecology. 2019 Dec 26;101(2):e02922. doi: 10.1002/ecy.2922 (PMC7027466; doi:10.1002/ecy.2922)
Supplement: Supplementary file 3 [file ECY-101-e02922-s003.pdf]

Supporting Information. Lauren G. Shoemaker*, Lauren L. Sullivan*, Ian Donohue, Juliano S. Cabral, Ryan J. Williams, Margaret M. Mayfield, Jonathan M. Chase, Chengjin Chu, W. Stanley Harpole, Andreas Huth, Janneke HilleRisLambers, Aubrie R.M. James, Nathan J.B Kraft, Felix May, Ranjan Muthukrishnan, Sean Satterlee, Franziska Taubert, Xugao Wang, Thorsten Wiegand, Qiang Yang, Karen C. Abbott. 2019. Integrating the underlying structure of stochasticity into community ecology. *Ecology.*

* Denotes equal contribution and corresponding authors

Appendix S3: Incorporating both demographic and environmental stochasticity in a single framework

**Model description**	**How demographic stochasticity is incorporated**	**How environmental stochasticity is incorporated**	**References**
Beverton-Holt *(Figs. 2-4)*	Poisson distribution of population sizes around the mean predicted by the deterministic model *(Eqns. 2,4,6,8)*	Temporally autocorrelated random variable that alters population size via the term $N_t\zeta\sigma_t$ with $\sigma_t = a\sigma_{t-1} + b\phi_t$. Parameters modify the magnitude (ζ) and autocorrelation (a) of the stochasticity. *(Eqns. 3,4,7,8)*	(Beverton and Holt 1957, Ripa and Lundberg 1996, Shoemaker and Melbourne 2016)
Beverton-Holt + seed bank *(Fig. 5)*	none	Temporally autocorrelated random variable that alters the germination rate $g_{i,t}$ = g_i+$\zeta_i\sigma_{i,t}$, with independent $\sigma_{i,t} = a\sigma_{i,t-1} + b\phi_{i,t}$ for each species i. *(Eqn. 9)*	(Beverton and Holt 1957, Ripa and Lundberg 1996, Levine and HilleRisLambers 2009)
Ricker *(Appendix S4, Fig. S1)*	none	Same as Beverton-Holt model. *(Appendix S4)*	(Ricker 1954, Ripa and Lundberg 1996)
Rosenzweig-MacArthur *(Figs. 6A,6B)*	none	Same as Beverton-Holt model with independent $\sigma_{t,n}$ and $\sigma_{t,p}$ for prey and predator. We use $a = 0$ (temporally uncorrelated stochasticity) in this examples. *(Appendix S5, Eqn. 1,2)*	(Rosenzweig and MacArthur 1963)
Freedman-Wolkowicz *(Fig. 6C)*	none	Same as Rosenzweig-MacArthur model. *(Appendix S5, Eqn. 3,4)*	(Freedman and Wolkowicz 1986)

Table S1: Description of the models used throughout the manuscript and how both demographic and environmental stochasticity are incorporated.

References:

Beverton, R., and S. J. Holt. 1957. On the dynamics of exploited fish populations. Ministry of Agriculture, Fisheries and Food, London, UK.

Freedman, H., and G. Wolkowicz. 1986. Predator-prey systems with group defence: The paradox of enrichment revisited. Bulletion of Mathematical Biology 48:493–508.

Levine, J. M., and J. HilleRisLambers. 2009. The importance of niches for the maintenance of species diversity. Nature 461:254–257.

Ricker, W. E. 1954. Stock and recruitment. Journal of Fisheries Research Board of Canada 11:559–623.

Ripa, J., and P. Lundberg. 1996. Noise colour and the risk of population extinctions. Proceedings of the Royal Society B: Biological Sciences 263:1751–1753.

Rosenzweig, M. L., and R. H. MacArthur. 1963. Graphical representation and stability conditions of predator-prey interactions. The American Naturalist 97:209–223.

Shoemaker, L. G., and B. A. Melbourne. 2016. Linking metacommunity paradigms to spatial coexistence mechanisms. Ecology 97:2436–2446.
