# Supplementary material for: Integrating the underlying structure of stochasticity into community ecology
Source: Ecology. 2019 Dec 26;101(2):e02922. doi: 10.1002/ecy.2922 (PMC7027466; doi:10.1002/ecy.2922)
Supplement: Supplementary file 4 [file ECY-101-e02922-s004.pdf]

Supporting Information. Lauren G. Shoemaker*, Lauren L. Sullivan*, Ian Donohue, Juliano S. Cabral, Ryan J. Williams, Margaret M. Mayfield, Jonathan M. Chase, Chengjin Chu, W. Stanley Harpole, Andreas Huth, Janneke HilleRisLambers, Aubrie R.M. James, Nathan J.B Kraft, Felix May, Ranjan Muthukrishnan, Sean Satterlee, Franziska Taubert, Xugao Wang, Thorsten Wiegand, Qiang Yang, Karen C. Abbott. 2019. Integrating the underlying structure of stochasticity into community ecology. *Ecology.*

* Denotes equal contribution and corresponding authors

Appendix S4: Modeling environmental stochasticity in populations and communities with overcompensatory dynamics

Incorporating overcompensatory dynamics and environmental stochasticity:

We replace the Beverton-Holt community model that incorporates environmental stochasticity with a Ricker model, allowing us to examine the interplay between population density dependence (i.e. overcompensatory dynamics in this case where growth rates respond more strongly than is needed to compensate for changes in environmental condition, causing an over- or undershoot of the carrying capacity and often oscillatory dynamics) and the structure of the autocorrelation in environmental stochasticity.

In this model,

$$N_{i,t+1} = N_{i,t}e^{[r_i(1-\sum_{j=1}^{S}\alpha_{ij}N_{j,t})+\zeta\sigma_{t+1}]},$$

where r is the intrinsic growth rate, S is the number of species in the regional species pool and α_{ij} is the pairwise competition coefficient of species j on species i (intraspecific competition is then considered when $j = i$). As in the Beverton-Holt model, ζ controls the magnitude of the effect of environmental stochasticity on population dynamics at each time interval. The time-series of environmental stochasticity is defined such that $\sigma_{t+1} = a\sigma_t + b\phi_t$ where $\sigma_0 = 0$ and b scales the magnitude of noise $\phi_t \sim Normal(0,1)$. We set $b = (1 - a^2)^{0.5}$ such that $var(\sigma)$ is the same for all a values (Ripa and Lundberg 1996). For populations, we examine overcompensatory dynamics (i.e. $r = 2.1$) with and without autocorrelation of environmental stochasticity ($a = 0$ vs. $a = 0.9$).

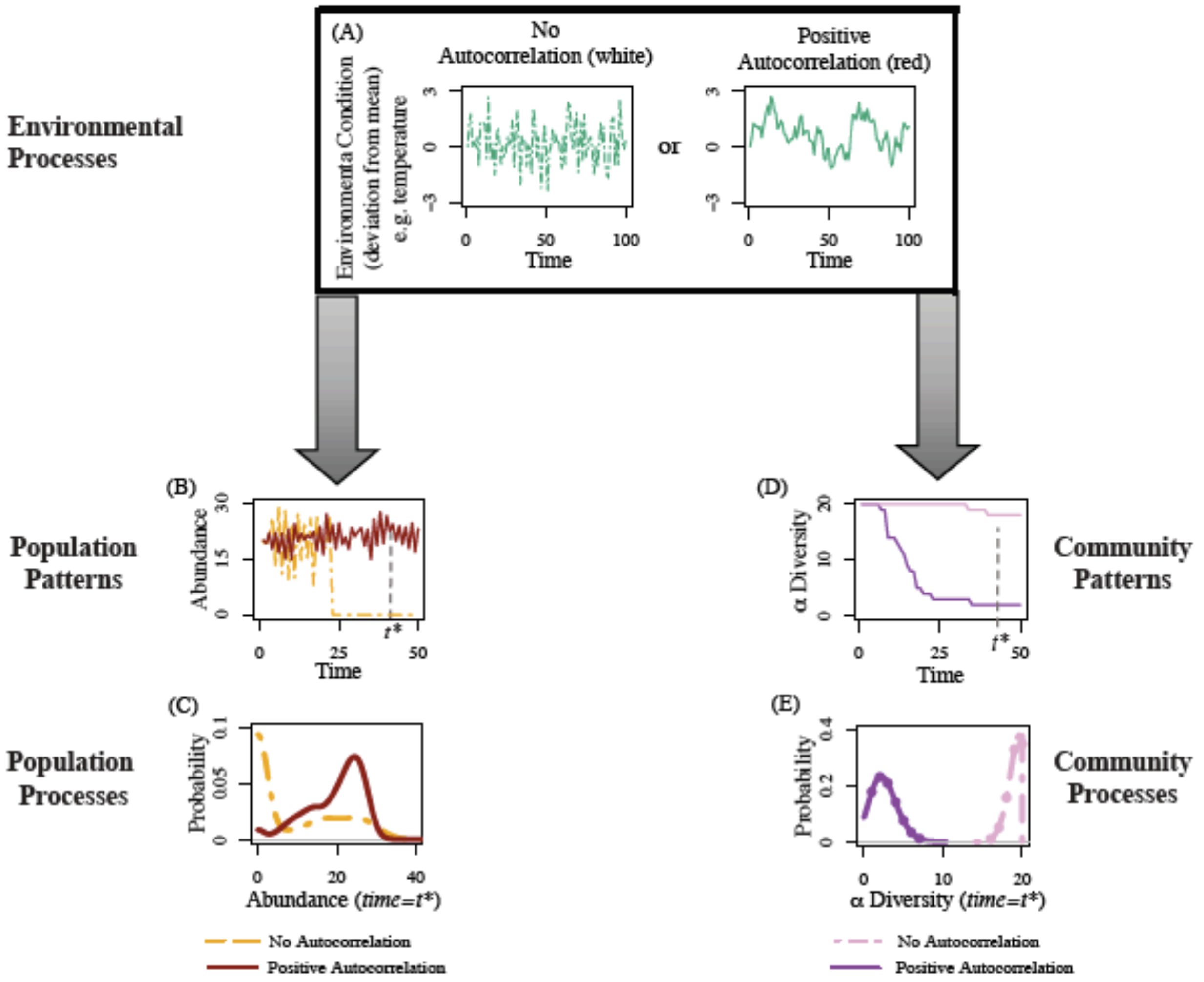

Figure S1. The autocorrelation in environmental stochasticity (A) alters population and community patterns ($a = 0$ for white noise; $a = 0.9$ for red noise and $\zeta = 0.25$). In overcompensatory populations, increasing the strength of positive autocorrelation in environmental stochasticity dampens population fluctuations (B) and therefore decreases the probability of extinction (C). Population model parameters are $r = 2.1$ and $\alpha = 0.05$. In communities where species respond independently to stochastic perturbations, increasing the strength of positive autocorrelation in environmental stochasticity decreases diversity (D and E). Community model parameters are $r_i \sim Uniform(1.4, 1.6)$ and $\alpha_{ij} = 0.01$ for all i, j.

Works Cited:

Ripa, J., and P. Lundberg. 1996. Noise colour and the risk of population extinctions. Proceedings of the Royal Society B: Biological Sciences 263:1751–1753.
