# Supplementary material for: Integrating the underlying structure of stochasticity into community ecology
Source: Ecology. 2019 Dec 26;101(2):e02922. doi: 10.1002/ecy.2922 (PMC7027466; doi:10.1002/ecy.2922)
Supplement: Supplementary file 5 [file ECY-101-e02922-s005.pdf]

Supporting Information. Lauren G. Shoemaker, Lauren L. Sullivan, Ian Donohue, Juliano S. Cabral, Ryan J. Williams, Margaret M. Mayfield, Jonathan M. Chase, Chengjin Chu, W. Stanley Harpole, Andreas Huth, Janneke HilleRisLambers, Aubrie R.M. James, Nathan J.B Kraft, Felix May, Ranjan Muthukrishnan, Sean Satterlee, Franziska Taubert, Xugao Wang, Thorsten Wiegand, Qiang Yang, Karen C. Abbott. 2019. Integrating the underlying structure of stochasticity into community ecology. *Ecology.*

Appendix S5: Model Descriptions for Box 2

Panels (A) and (B) in Figure 6 of the main text, referenced in Box 2, were created using the Rosenzweig-MacArthur model (Rosenzweig & MacArthur 1963) with environmental stochasticity added:

$$\frac{dN}{dt} = rN\left(1 - \frac{N}{K}\right) - \frac{mNP}{1+mhN} + N\zeta_n\sigma_{t,n} \tag{S1}$$

$$\frac{dP}{dt} = \frac{cmNP}{1+mhN} - dP + P\zeta_p\sigma_{t,p} \tag{S2}$$

where N is the prey population density, P is the predator density, r is the prey population's intrinsic growth rate, K is its carrying capacity, m is the maximum predation rate, h is the handling time, c is the predator efficiency, and d is the predator's death rate. Environmental stochasticity is added via the final term in each equation, as described for the Beverton-Holt model in the main text. We use $a = 0$ for both predator and prey, which makes $\sigma_{t,n}$ and $\sigma_{t,p}$ uncorrelated *Normal*(0,1) random variables. We used $r = 0.2$, $K = 82$, $m = 0.02$, $h = 1$, $c = 1$, $d = 0.4$, $\zeta_n = 0.01$, and $\zeta_p = 0.03$ for the stochastic trajectories (yellow, red) shown in Figure 6. Deterministic trajectories (black, gray) used the same parameter values except $\zeta_n = \zeta_p = 0$.

Panels (C) and (D) in Figure 6 were generated from the Freedman-Wolkowicz model (Freedman & Wolkowicz 1986) with environmental stochasticity added:

$$\frac{dN}{dt} = rN\left(1 - \frac{N}{K}\right) - \frac{mNP}{N^2+m(N+1)} + N\zeta_n\sigma_{t,n} \tag{S3}$$

$$\frac{dP}{dt} = \frac{cmNP}{N^2+m(N+1)} - dP + P\zeta_p\sigma_{t,p}. \tag{S4}$$

Parameter values for the stochastic realizations were $r = 1$, $K = 2.5$, $m = 4.5$, $c = 5$, $d = 2.5$, $\zeta_n = 0.01$, and $\zeta_p = 0.25$. Deterministic trajectories used the same values except $\zeta_n = \zeta_p = 0$.

References

Freedman, H., and G. Wolkowicz 1986. Predator-prey systems with group defence: The paradox of enrichment revisited. Bulletin of Mathematical Biology 48:493–508.

Rosenzweig, M. L., and R. H. MacArthur 1963. Graphical representation and stability conditions of predator-prey interactions. The American Naturalist 97:209–223.
